# Calycosin protects from histopathological and ultrastructural changes in ethanol-induced liver injury and exhibits predicted binding affinity to TNF-α/NF-κB/HIF-1α proteins

**DOI:** 10.3389/fphar.2026.1860075

**Published:** 2026-08-06

**Authors:** Abeer A. K. Mohamed, Yasmine G. Sabry, Amr Elmistekawy, Tahani Saaedi, Isra Omar, Sawsan A. Zaitone, Noura Moustafa Mohamed, Dina M. Khodeer, Amira H. Eltrawy

**Affiliations:** 1 Department of Human Anatomy, College of Medicine, Jazan University, Jazan, Saudi Arabia; 2 Histology and Cell Biology Department, Faculty of Medicine, Suez Canal University, Ismailia, Egypt; 3 Physiology Department, Faculty of Medicine, Ain Shams University, Cairo, Egypt; 4 Department of Internal Medicine, Gastroenterology Division, Faculty of Medicine, Al-Azhar University, Cairo, Egypt; 5 Department of Pharmacology and Toxicology, College of Pharmacy, Taibah University, Madinah, Saudi Arabia; 6 Health and Life Research Center, Taibah University, Madinah, Saudi Arabia; 7 Department of Clinical Medicine, College of Medicine, Almaarefa University, Riyadh, Saudi Arabia; 8 Royal College of Physicians of Ireland, Dublin, Ireland; 9 Department of Pharmacology and Toxicology, Faculty of Pharmacy, University of Tabuk, Tabuk, Saudi Arabia; 10 Department of Basic Sciences, Faculty of Medicine, Princess Nourah bint Abdulrahman University, Riyadh, Saudi Arabia; 11 Department of Pharmacology, College of Medicine, Imam Mohammad Ibn Saud Islamic University (IMSIU), Riyadh, Saudi Arabia; 12 Department of Anatomy and Embryology, Faculty of Medicine, Alexandria University, Alexandria, Egypt

**Keywords:** calycosin, ethanol-induced liver injury, histopathology, rat, TNF-α/NF-κB/HIF-1α signaling

## Abstract

**Background/objectives:**

Liver fibrosis is the consequence of the wound-healing response of the liver to frequent injury. Calycosin is a flavonoid, which has been documented for its hepatoprotective properties. The aim of this study was to evaluate the hepatoprotective action of calycosin in a model of ethanol-induced liver injury (EILI) in rats. For mechanistic insights, we aimed to measure tumor necrosis factor-α (TNF-α), nuclear factor-κB (NF-κB), and hypoxia-inducible factor-1 alpha (HIF-1α) protein levels in liver samples and evaluate the ability of calycosin to bind these pathologic molecules.

**Methods:**

Network pharmacology and molecular docking were performed for discovering the interacting target proteins. Forty male rats were assigned into five groups: 1) the vehicle, 2) the calycosin per se group, 3) the EILI control group, and 4) and 5) the EILI + calycosin (5 or 10 mg/kg) groups. The liver homogenates were used for ELISA measurement of TNF-α, NF-κB, and HIF-1α. Furthermore, liver specimens were used for histopathological and ultrastructural investigations.

**Results:**

The network pharmacology study confirmed the role of TNF-α/NF-κB/HIF-1α signaling in EILI, and molecular docking explored the possible interaction between calycosin and these three molecules (docking score = −7.6, −7, and −7.6 kcal/mol). The rat study showed that calycosin was able to attenuate histopathological and ultrastructural changes in rat livers, reduce collagen accumulation, and prevent the increases shown in liver enzyme activities (2.17-fold for ALT and 2.26-fold for AST).

**Conclusion:**

Thus, these integrated *in silico* and *in vivo* studies confirmed the hepatoprotective effect of calycosin against the EILI rat model and provided a mechanistic insight through TNF-α/NF-κB/HIF-1α signaling. Further studies are warranted to fully explore the protective mechanism of calycosin in EILI.

## 1 Introduction

Chronic liver disease is a worldwide health problem, and it causes approximately two million deaths annually (Asrani et al., 2019). Hepatic fibrosis is the consequence of the wound-healing response of the liver to frequent injury (Svegliati-Baroni et al., 2008). This activity is accompanied by inflammation and a partial deposition of matrix proteins (MPs). When there is a persistent hepatic injury, liver regeneration fails, and the substitution of hepatocytes takes place with abundant MPs, comprising fibrillar collagen. The fibrous material distribution relies on the causative factor of liver injury (Pinzani, 2000). The composition of the MP, during liver fibrosis, alters from collagen IV, collagen VI, glycoproteins, and proteoglycans into collagen I, collagen III, and fibronectin (Brown et al., 2006).

Modern therapeutic choices for chronic hepatic diseases are inadequate, and current medicines may lack effectiveness. The efficacy of existing medicines such as interferons and corticosteroids is inconsistent, leading to the risk of unwanted adverse effects, as well as excessive costs. Therefore, researchers are struggling to identify applicable medications with minimal adverse effects for addressing the challenges associated with the unreliability of modern pharmaceutical agents. Some drugs can reduce the accumulation of scar tissue in experimental animal models of chronic liver injuries. Antioxidants are the most favorable drugs and are promising in this field (Abd Rashid et al., 2021).

Herbal remedies were identified to be hepatoprotective and were commonly utilized to treat illnesses (Abou Seif, 2016). Plant phytochemicals are a distinctive class of secondary metabolites that are known to play a role in protection from liver diseases. Traditional Chinese medicine has developed new therapies for liver fibrosis. Isoflavones are receiving attention due to their ability to prevent liver diseases, atherosclerosis, adiposity, cardiovascular disease, and hyperlipidemia (Kim and Kang, 2012). Calycosin (7,3′-dihydroxy-4′-methoxyisoflavone, PubChem CID: 5280448) is one of the main bioactive compounds derived from the root of Radix Astragali [the dry root of *Astragalus propinquus* Schischkin (Fabaceae)]. Calycosin produces favorable protective properties against acute liver injury (Chen et al., 2015) and mitigates triglyceride (TG) accumulation in a model of non-alcoholic steatohepatitis and fibrosis through activation of the farnesoid X receptor (Duan et al., 2017). In some studies, the authors highlighted that calycosin mitigated the hepatic steatosis model via modulation of some of the key regulatory factors (Cheng et al., 2020). However, concrete therapeutic targets and mechanisms of calycosin in ethanol-induced liver injury (EILI) are still to be determined.

To the best of our knowledge, the action of calycosin as a protective agent against EILI has not been reported previously. This study was conducted to determine whether calycosin can provide anti-inflammatory activity in a male rat model of EILI. We used *in silico* methods and *in vivo* verification using serological, histological, immunohistochemical, and ultrastructural methods.

In this study, we aimed to evaluate the putative hepatoprotective effect of calycosin in a rat model of EILI using serological, histological (routine staining and special stains), immunohistochemical, and ultrastructure methods. For mechanistic insights, we aimed to measure tumor necrosis factor-α (TNF-α), nuclear factor-κB (NF-κB), and hypoxia-inducible factor-1 alpha (HIF-1α) protein levels in liver tissue samples and evaluate the ability of calycosin to bind to these pathologic molecules and downregulate the inflammatory status by assessing some downstream inflammatory cytokines in the liver tissues.

## 2 Materials and methods

### 2.1 Network pharmacology analysis

This computational analysis was performed according to previously published protocols and used similar tools, such as the STRING and KEGG databases (Zaitone S. et al., 2026; Zaitone et al., 2026). Accession dates, references, and platforms’ links are listed in Table 1, and all resulting targets from the upcoming databases were considered for subsequent analysis, with no specific filtration system to preclude any possible bias, which may be linked to arbitrary cutoff values, especially when there are no established guidelines that govern the optimal cutoff or threshold for the selection process. In addition, early exclusion of targets with low probability might lead to excluding targets with an essential role in the protein–protein interaction (PPI) network according to their centrality (Supplementary file 1).

**TABLE 1:** List of used databases with their accession dates and URLs.

Database	Accession date	URL
PubChem	5/1/2026	https://pubchem.ncbi.nlm.nih.gov/compound/5280448
Swiss Target Prediction	5/1/2026	https://www.swisstargetprediction.ch/
SuperPred	5/1/2026	https://prediction.charite.de/
Comparative Toxicogenomic Database (CTD)	5/1/2026	https://ctdbase.org/
GeneCards	6/1/2026	https://www.genecards.org/
Open Target Platform (OTP)	6/1/2026	https://platform.opentargets.org/
UniProt	7/1/2026	https://www.uniprot.org/
SRplot tool	8/1/2026	https://www.bioinformatics.com.cn/srplot
STRING 12.0	8/1/2026	https://string-db.org/
ShinyGO 0.85.1	9/1/2026	https://bioinformatics.sdstate.edu/go/
RCSB Protein Data Bank (RCSB PDB)	9/1/2026	https://www.rcsb.org/

#### 2.1.1 Obtaining targets associated with calycosin and EILI

The SMILE of “Calycosin, COC1 = C(C=C(C=C1)C2 = COC3 = C(C2 = O)C=CC(=C3)O)O″ was retrieved from PubChem and then entered into the SwissTargetPrediction and SuperPred databases using *Homo sapiens* as the target species to obtain calycosin’s targets, with a probability> 0. In addition, ethanol targets were gathered from the SuperPred database and Comparative Toxicogenomics Database (CTD).

After that, GeneCards and Open Target Platform (OTP) were accessed for collecting targets linked to liver fibrosis with a relevance score >0. The intersection between the targets of ethanol and liver fibrosis was considered the disease-associated targets.

Then, the three lists were sent to an online SRPlot tool to identify and visualize the overlapping targets among calycosin and EILI.

#### 2.1.2 Building protein–protein interaction (PPI) network

The overlapping targets were presented in their official gene symbols with reference to HGNC nomenclature using the UniProt database. Then, these targets were entered into the STRING database for constructing a PPI network, with “*Homo sapiens*” specified as the target species and the default medium confidence score set to 0.4; the resulting network was subsequently imported into Cytoscape 3.10.2 for analysis.

#### 2.1.3 Screening core targets of calycosin for ethanol-induced liver injury

CytoNCA plug-in was utilized to rank the overlapping targets based on their topological parameters: degree, betweenness, closeness, and eigenvector. Further experimental analysis was applied on selected targets among the top 10 targets for each metric, guided by previous literature reviews.

The conservation among homologues proteins in human race and rats was evaluated using the UniProt database as performed in previous studies (Zaitone S. A. et al., 2026; Zaitone S. et al., 2026). UniProt verified rat orthologs of the key human targets, with a matching score 10 for TNF-α, HIF-1α, and 8 for NF-κB, as listed in Supplementary file 2.

#### 2.1.4 Enrichment analysis

To predict the possible therapeutic pathways and biological processes modulated by calycosin in ethanol-induced liver injury, the overlapped targets, in their official gene symbols, were subjected to enrichment analysis using the ShinyGo database, with *Homo sapiens* as the target species. The upper 20 terms in molecular functions (MF), biological processes (BP), cellular components (CC), and KEGG pathways were visualized, ranked according to the FDR values (FDR cutoff <0.05), and sorted by their fold enrichment.

### 2.2 Molecular docking analysis

To explore the interaction between calycosin and potential therapeutic targets included in the disease, hub targets from network pharmacology were considered for docking analysis via PyRx 0.8. First, the 3D structure of the ligands was obtained in their SDF formats from PubChem (PubChem CID of calycosin: 5280448, SPD304: 5327044, sesamin: 72307, and pycnidione: 135454124); then, they were modified into PDB format using Open Babel. Next, we downloaded the protein structures of the selected targets from the RCSB Protein Data Bank (RCSB PDB), TNF: 2AZ5, NF-κB: 1NFK, and HIF-1α: 4ZPR, and prepared using the Biovia Discovery Studio visualizer, with removing water molecules and converting it to be a macromolecular target in PyRx.

Finally, PyRx software was used to perform blind docking, with an exhaustiveness of 8, using the entire protein as a grid center to avoid arbitrary bias in screening the possible binding sites. Since blind docking was adopted with the aim of comparing, rather than optimizing, affinity, classical redocking and RMSD calculation were considered not applicable, particularly when no co-crystalized ligands are available, and the interpretation was demonstrated as supportive evidence to complement the network pharmacology findings and experimental validation, rather than as definitive proof of binding affinity.

In addition, the affinity scores in Kcal/mol were relatively measured, with the least value being considered the best mode, followed by a visual analysis using the Biovia Discovery studio visualizer. The docking score for each mode was compared to a reference inhibitory ligand based on previous literature reviews. For instance, SPD304 is a TNF-α inhibitor (Moawadh, 2023), Sesamin inhibits NF-κB (Maslikah et al., 2025), and pycnidione binds competitively to HIF-1α (Reda et al., 2026).

### 2.3 The *in vivo* rat study

#### 2.3.1 Chemicals and drugs

Ethanol in analytical grade was purchased from Elnasr Company for Intermediate Chemicals (Cairo, Egypt) and used and diluted with distilled water to reach a final concentration of 25%. Calycosin (C16H12O5, purity ≥98%, MWt = 284.26 g/mol) was purchased from Qingyang Inter-Healthcare Factory Co., Ltd (Qingyang City, Gansu Province, China) in a pure powdered form and suspended in 1% carboxymethylcellulose solution.

#### 2.3.2 Sample size calculation and experimental animals

To calculate the sample size, a previously published equation (Charan and Kantharia, 2013) was used, applying data from Chen et al. (2015). The effect size was 22.73, the estimated standard deviation was 15.236, and the sample size per group was calculated to equal 8 rats.

Hence, 40 healthy male adult Wistar rats were purchased from Mostafa Rashed Company. The range of rat body weight at the beginning of the study was 118–133 g. Male rats were used to reduce biological variability associated with estrus cycle-associated hormonal fluctuation and to maintain consistency with the established protocol for ethanol-induced liver injury (Safer et al., 2015; El-Sisi et al., 2017). Rats were randomized using the simple random number table method based on body weight stratification to avoid unbiased selection of rats. Rats were kept in clean plastic cages in groups of four, with free access to food and water. Strict care and hygiene were maintained in a normal dark/light cycle. This study was conducted following approval from the Research Ethics Committee of the Faculty of Medicine, Suez Canal University (6269#), Ismailia, Egypt. The animal study complied with the ARRIVE 2.0 guidelines. All the needed precautions were taken to reduce animals’ suffering to the utmost extent possible. The rats welfare was monitored during the 7-week ethanol administration period (e.g., body weight monitoring and clinical observation every week). Early termination was planned if severe body weight loss (more than 25% of the original body weight) or severe sickness was observed.

#### 2.3.3 The study design

As demonstrated in Table 2, the rats were allocated in a random manner to five groups; each group consisted of eight male rats and was categorized as follows:

1. Double vehicle group: rats were gavaged with distilled water (instead of ethanol solution) twice a week (day 1 and day 2) at 9 a.m. and received a 1% CMC suspension via oral gavage, five times per week (day 3 to day 7) at 3 p.m. for 7 weeks.
2. Calycosin per se group: rats were gavaged with distilled water (instead of ethanol) at 9 a.m. and received calycosin (10 mg/kg) via oral gavage (Elsherbiny et al., 2020), five times per week (day 3 to day 7) at 3 p.m. for 7 weeks.
3. EILI control group: rats received ethanol (25%, volume/volume) (1 mL/100 g/day, p. o.) via oral gavage, twice a week (day 1 and day 2) for 7 weeks in male rats (El-Sisi et al., 2017); this treatment was administered at 9 a.m., and the rats received a 1% CMC suspension via oral gavage, five times per week (day 3 to day 7) at 3 p.m.
4. EILI + calycosin (5 mg/kg): rats received ethanol as mentioned above at 9 a.m. and received calycosin (5 mg/kg) (Elsherbiny et al., 2020) by oral gavage five times per week (day 3 to day 7) at 3 p.m.
5. EILI + calycosin (10 mg/kg): rats received ethanol as mentioned above at 9 a.m. and calycosin (10 mg/kg) via oral gavage five times per week (day 3 to day 7), at 3 p.m.

**TABLE 2:** Description for the dose regimens in the study groups.

Number	Description	Day 1 and day 2 By oral gavage at 9 a.m	Day 3–day 7 By oral gavage at 3 p.m
Group 1	Double vehicle	Dist. Water (1 mL/100 g)	1% CMC suspension
Group 2	Calycosin per se	Dist water (1 mL/100 g)	Calycosin (10 mg/kg)
Group 3	EILI control	Ethanol (1 mL/100 g)	1% CMC suspension
Group 4	EILI + calycosin (5 mg/kg)	Ethanol (1 mL/100 g)	Calycosin (5 mg/kg)
Group 5	EILI + calycosin (10 mg/kg)	Ethanol (1 mL/100 g)	Calycosin (10 mg/kg)

#### 2.3.4 Rat scarification and sample collection

Rats were anesthetized with ketamine, and blood samples were withdrawn using a hematocrit tube from the retro-orbital plexus and then centrifuged at 1500 *g* for 10 min to obtain the serum. The serum samples were aliquoted into two tubes and stored at −80 °C. The whole liver was dissected and washed out of blood. The largest hepatic loop was dissected and fixed in 10% paraformaldehyde solution for 24 h, and then, paraffin blocks were prepared and used to obtain tissue specimens used for immunohistochemical and histological staining. The remaining liver lobes were dissected, immediately frozen at −80 °C, and stored until subsequent biochemical assays.

#### 2.3.5 Assessment of serum liver enzyme activities and ELISA measurements

Enzymatic colorimetric assay kits (SPINREACT, Girona, Spain) were employed for the measurement of the liver function. Serum aspartate aminotransferase (AST) and alanine aminotransferase (ALT) activities were measured using a spectrophotometer according to the manufacturer’s instructions. Furthermore, frozen liver samples were homogenized in phosphate buffered saline (PBS), and the homogenate was centrifuged at 1500 *g* for 10 min to obtain clear homogenates and used for determining the following markers using MyBioSource ELISA kits: TNF-α (MBS355371, sensitivity is less than 1 pg/mL, with a detection range of 15.6–1000 pg/mL), HIF-1α (MBS728803, sensitivity is 0.1 pg/mL, with a detection range of 25–500 pg/mL), and NF-κB (MBS722386, sensitivity is 0.1 ng/mL, with a detection range of 0.5–10 ng/mL), with intra-assay CV less than 10%. The sample dilution factor was 50, and the assays were performed in a blinded manner.

We used the primary/detection antibodies following each kit instructions for obtaining optimal curves. Furthermore, the same controls and reagents were used for all assays. To ensure technical consistency and reduce intra-assay variability, calibrated micropipettes, stable incubation temperature, and firm incubation timings were set. Finally, we recorded the readings of each cell at the indicated wave length, and the results were normalized to the weight of the samples.

#### 2.3.6 Histopathological and immunohistochemical evaluation

The liver specimens were dissected from the rats and cut into 4-mm-thick tissue blocks. Then, the specimens were fixed in 10% buffered formalin solution for 24 h, dehydrated in ascending grades of alcohol (70%, 90%, 100%, 100%, and 100% for 3 h each), cleared in xylene, infiltrated using melted wax at 65 °C, and embedded in liquid paraffin wax to form tissue blocks.

Each tissue block was cut into six serial sections; each of them was 4-µm thick. Two sections were stained with hematoxylin and eosin (H&E) staining, and two sections were stained with Masson’s trichrome (MT) stain. The other two sections were mounted on poly-L-lysine-coated slides for immunohistochemical staining. The sections were deparaffinized, rehydrated, and incubated in H_2_O_2_; then, the slides were rinsed in PBS, and for antigen retrieval, the slides were placed in a microwave oven containing citrate buffer at pH = 6. The slides were incubated in 10% normal goat serum and a serum-free protein block (10 min).

We used an antibody for α-smooth muscle actin (SMA) protein incubation with a rabbit polyclonal primary antibody (diluted 1:500, GTX100034, GeneTex) at room temperature. The Bio SB Detection System (Santa Barbara, CA 93117, United States) was used to detect the resulting immune complex. The secondary antibody and streptavidin–peroxidase complex were used. The sections were counterstained in hematoxylin and rinsed gently in tap water. Finally, the slides were dehydrated, cleared in xylene, cover-slipped, and then examined using a light microscope, Olympus CX31.

#### 2.3.7 Histopathological assessment

The criterion for the classification of the degree of hepatocyte affection in H&E sections was as follows: histopathological changes were assessed using a semi-quantitative method. Each hepatic specimen was given a score according to the severity of the pathologic manifestations as listed in Table 3 (Batts and Ludwig, 1995). The assessor was blinded to the study groups. In addition, quantitative measurements were conducted to assess the area% of the green color in Masson’s trichrome sections and the α-SMA protein area in the immunostained liver sections imaged at ×400 magnification using the ImageJ image analysis system (NIH, United States).

**TABLE 3:** The criterion for the classification of the degree of affection of hepatocytes.

Degree of affection	Criteria
No affection (0)	No morphological changes
Mild affection (1)	Swollen cells with pale cytoplasm (cloudy swelling)
Moderate affection (2)	Vacuolated cytoplasm (hydropic degeneration)
Severe affection (3)	Nuclear change with cytoplasmic affection (necrosis)

In brief, we performed unmixing of brightfield images into channels representative of every dye’s absorbance. Following this step, we changed images of brown color of immunostaining or the color of the Masson’s trichrome stain into gray by using the threshold tool to determine the structure area. Finally, in contrast with the white background, we determined the area percentage of the targeted stained structures. This is a known method of quantification of the stained area of each field (Alhaj et al., 2015). We analyzed images from five regularly spaced fields from two sections from each rat, and the average value for each group was calculated.

#### 2.3.8 Transmission electron microscopic examination

The specimens (1 mm^3^) were fixed in a solution of 2.5% glutaraldehyde at 4 °C for 24 h. They were then post-fixed in osmium tetroxide (2%) for 1 h, followed by dehydration and resin embedding. We cut ultrathin sections and stained them with uranyl acetate and lead citrate (Sigle, 2005). The grids and photographs were examined using a transmission electron microscope (JEOL-EX 1010 TEM). The TEM images were evaluated by a blinded observer.

### 2.4 Statistical analysis

The mean and standard deviation were calculated for each variable. For analyzing the continuous quantitative data, normal distribution was confirmed using the Shapiro–Wilk test for each endpoint. Second, the p-value was determined using the one-way ANOVA test and Tukey’s *post-hoc* test. For the histopathology score, an ordinal scale for the degree of affection was used, and median values were presented and compared with a non-parametric ANOVA test followed by pair-wise comparison using the Dunn’s test. A p-value less than 0.05 for a difference was considered statistically significant.

## 3 Results

### 3.1 Network pharmacology analysis

**FIGURE 1:**
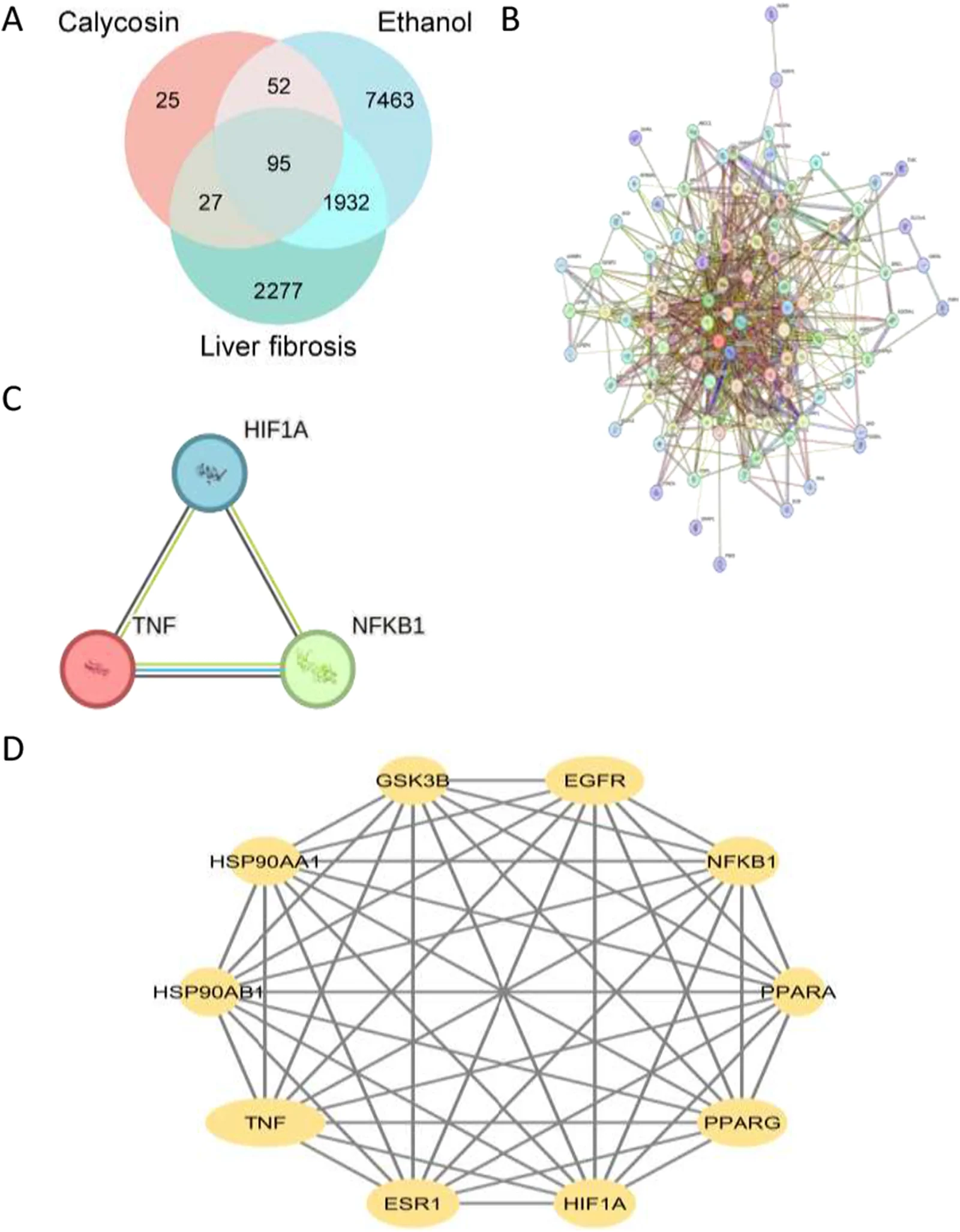
Network pharmacology analysis of targets linked to calycosin and ethanol-induced liver fibrosis. **(A)** Venn diagram of calycosin and disease-associated targets showing 95 overlapped targets. **(B)** PPI network showing the targets in common with a confidence level >0.4 (95 nodes and 634 edges) from the STRING database. **(C)** PPI network of the three hub targets which validated experimentally with predicted associations among them. **(D)** Top 10 targets were ranked based on their values analyzed using Cytoscape.

To reveal the possible targets of calycosin in curing EILI, a network pharmacology analysis was performed (Figure 1). First, 199 targets related to calycosin were identified using Swiss Target Prediction and SuperPred, while targets linked to liver fibrosis was considered the intersection between ethanol targets and liver fibrosis targets from CTD, SuperPred, GeneCards, and OTP. Output numbers from each database are listed in Table 4, and detailed targets are listed in Supplementary Table 1.

**TABLE 4:** Output numbers from each database.

Drug/Disease	Database	Target numbers	Target numbers after removal of repetitions
Calycosin	Swiss Target Prediction	100	199
	SuperPred	111	
Ethanol	SuperPred	93	9542
	CTD	9511	
Liver fibrosis	GeneCards	2934	4331
	OTP	2600	

Afterward, a Venn diagram was constructed using SRplot, and 95 overlapped targets were recognized (Figure 1A). Following that, the PPI network sourced from STRING involved 95 nodes and 634 edges (Figure 1B), with focusing on the predicted association among the three experimentally validated targets in Figure 1C. The top 10 hub targets of calycosin involved in managing hepatic fibrosis are demonstrated in Figure 1D. TNF-α, HIF-1α, and NF-κB were selected for further experimental validation based on their topological values and guided by previous literature reviews on their roles in this disease model. Notably, TNF-α showed the highest number of associations, linked to 65 proteins in the network. Furthermore, HIF-1α and NF-κB were associated with 44 and 40 proteins, respectively. Top targets with their relevant topological values are listed in Table 5, and common targets are listed in Supplementary Table 2.

**TABLE 5:** Topological parameters of top 10 targets.

Rank	Target name	Degree	Eigenvector	Betweenness	Closeness
1	TNF-α	65	0.271532	2007.622	0.752
2	EGFR	53	0.254217	872.9596	0.681159
3	ESR1	50	0.244358	993.301	0.676259
4	PPARG	49	0.242163	640.73	0.661972
5	HIF-1α	44	0.230191	525.4567	0.635135
6	NF-κB	40	0.22857	251.1893	0.618421
7	HSP90AA1	39	0.22126	247.0903	0.614379
8	GSK3B	38	0.210246	535.2745	0.61039
9	HSP90AB1	33	0.193604	176.0292	0.5875
10	PPARA	29	0.171305	303.0155	0.569697

**FIGURE 2:**
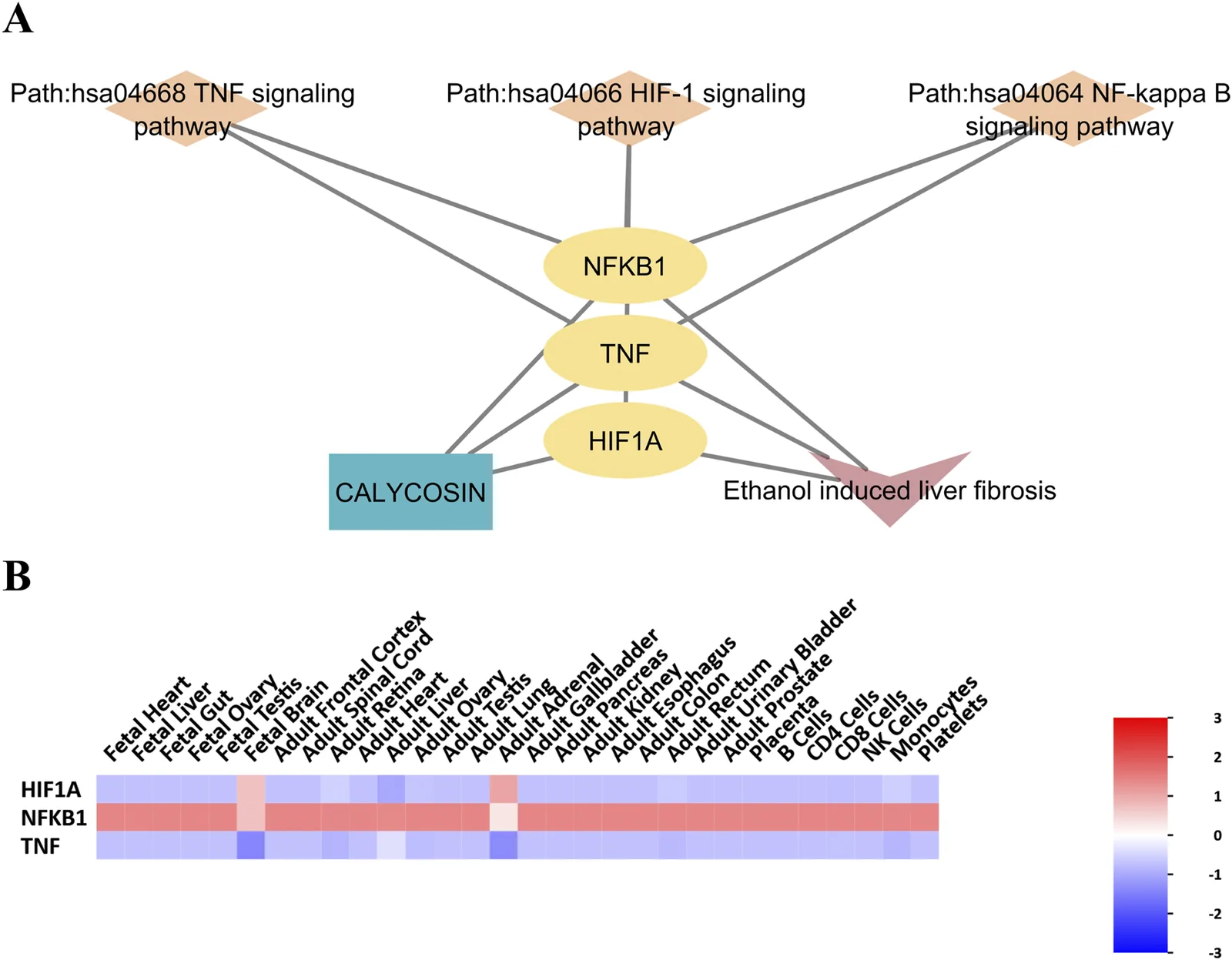
**(A)** Drug–disease–target–pathway network. Drug: rectangle, disease: V-shape, target proteins: ellipse, and pathways: diamond. **(B)** Heatmap showing the gene expression pattern of calycosin targets. 3: most expressed gene (intense red color), −3: least expressed gene (intense blue color).

Furthermore, a drug–disease–target–pathway network was constructed using Cytoscape (Figure 2A). The tissue-specific expression patterns of the core calycosin-associated targets—HIF-1α, NF-κB, and TNF-α—were characterized using heatmap visualization (Figure 2B). The heatmap displays normalized gene expression values ranging from −3 (lowest expression; deep blue) to +3 (highest expression; deep red) across a broad panel of human tissues.

NF-κB displayed the highest and most widespread basal expression across nearly all examined tissues and immune cell types, including consistently elevated levels in both fetal and adult liver, underscoring its broad regulatory role. HIF-1α showed moderate and tissue-dependent expression, with relatively higher levels in metabolically active organs. In contrast, TNF-α exhibited generally low baseline expression, with slight elevation in immune-related compartments and adult liver tissue. Together, these patterns indicate that NF-κB is the most broadly expressed target, while HIF-1α and TNF-α maintain more restricted and context-dependent expression profiles, supporting the central role of NF-κB in mediating calycosin’s biological effects.

**FIGURE 3:**
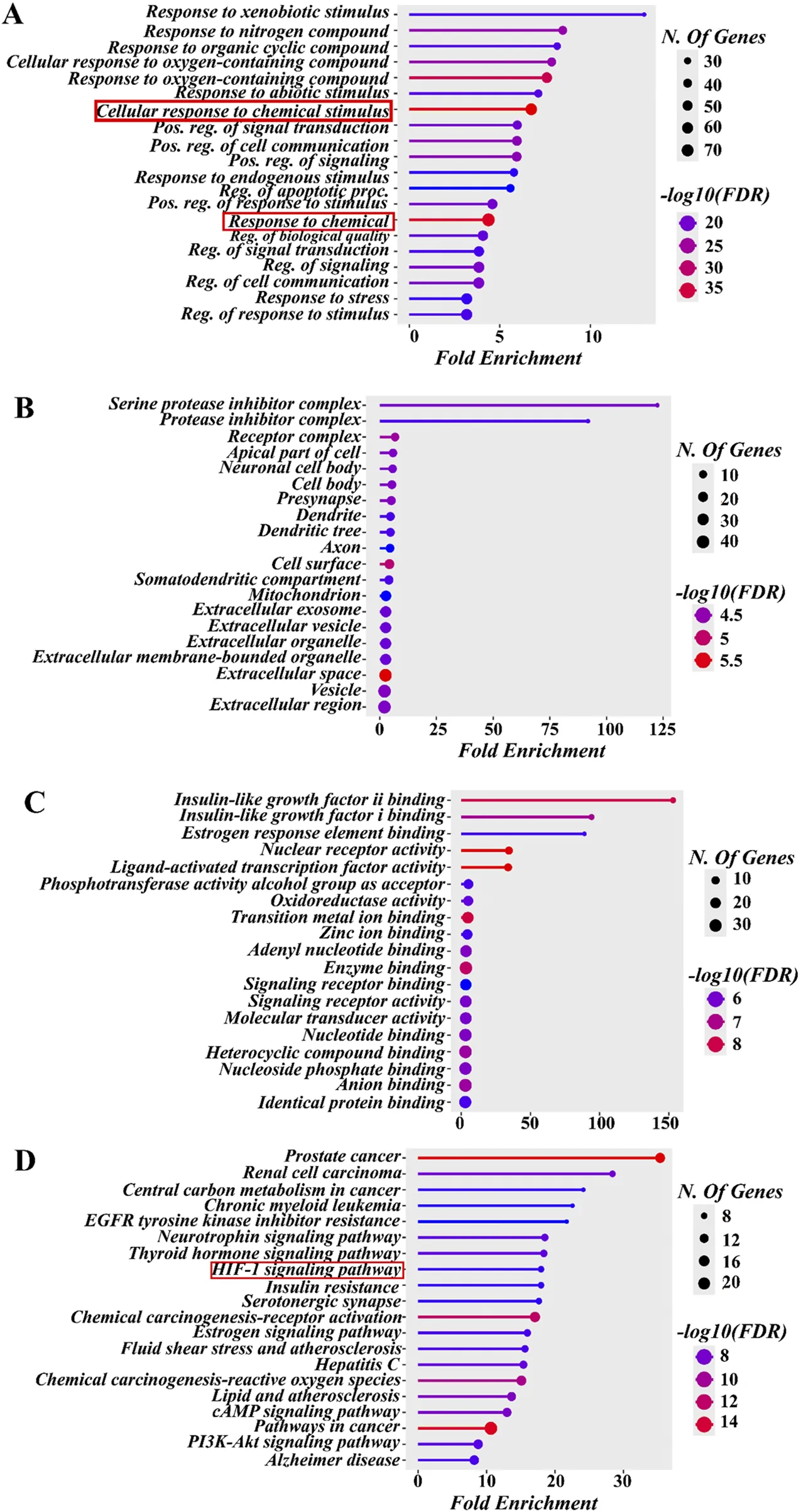
Enrichment analysis of 95 common targets between calycosin and ethanol-induced liver fibrosis using ShinyGO, FDR <0.05. Top 20 enrichment terms: **(A)** biological processes. **(B)** cellular components **(C)** molecular functions. **(D)** KEGG pathways.

To furnish a comprehensive explanation for the prospective action mechanisms for calycosin, an enrichment analysis was executed using the ShinyGO database. The exploration addressed BP, CC, MF, and KEGG pathways, yielding 1,938 statistically significant terms, consisting of 1,000 BP, 207 CC, 515 MF terms, and 216 KEGG pathways (Supplementary Table 3). Lollipop charts were implemented to illustrate the top 20 terms in each category (Figure 3).

The enrichment results showed that the targets of calycosin in treating liver fibrosis were chiefly enriched in biological processes, such as cellular response to chemical stimulus (GO: 0070887), response to chemical compounds (GO: 0042221), and response to oxygen-containing compounds (GO: 1901700), in addition to other biological processes. Moreover, they were enriched in cellular components, such as extracellular space (GO: 0005615), cell surface (GO: 0009986), and receptor complex (GO: 0043235), among others. Furthermore, the overlapping targets were found to exhibit molecular functions, such as nuclear receptor activity (GO:0004879), ligand-activated transcription factor activity (GO: 0098531), and transition metal ion binding (GO: 0046914). The KEGG outcomes reveal hepatoprotective effects of calycosin via many mechanisms, such as HIF-1α signaling pathway (Path: hsa04066), TNF-α signaling pathway (Path: hsa04668), and NF-κB signaling pathway (Path: hsa04064).

### 3.2 Molecular docking

For further validation of the mechanism of calycosin against hepatic fibrosis and to investigate the binding affinity to key targets from network pharmacology, it is accepted that the lower docking energy reflects stronger interaction between the drug and proteins. The docking results depict that calycosin can bind to the three target proteins, indicating moderate binding ability when compared with their inhibitors.

**FIGURE 4:**
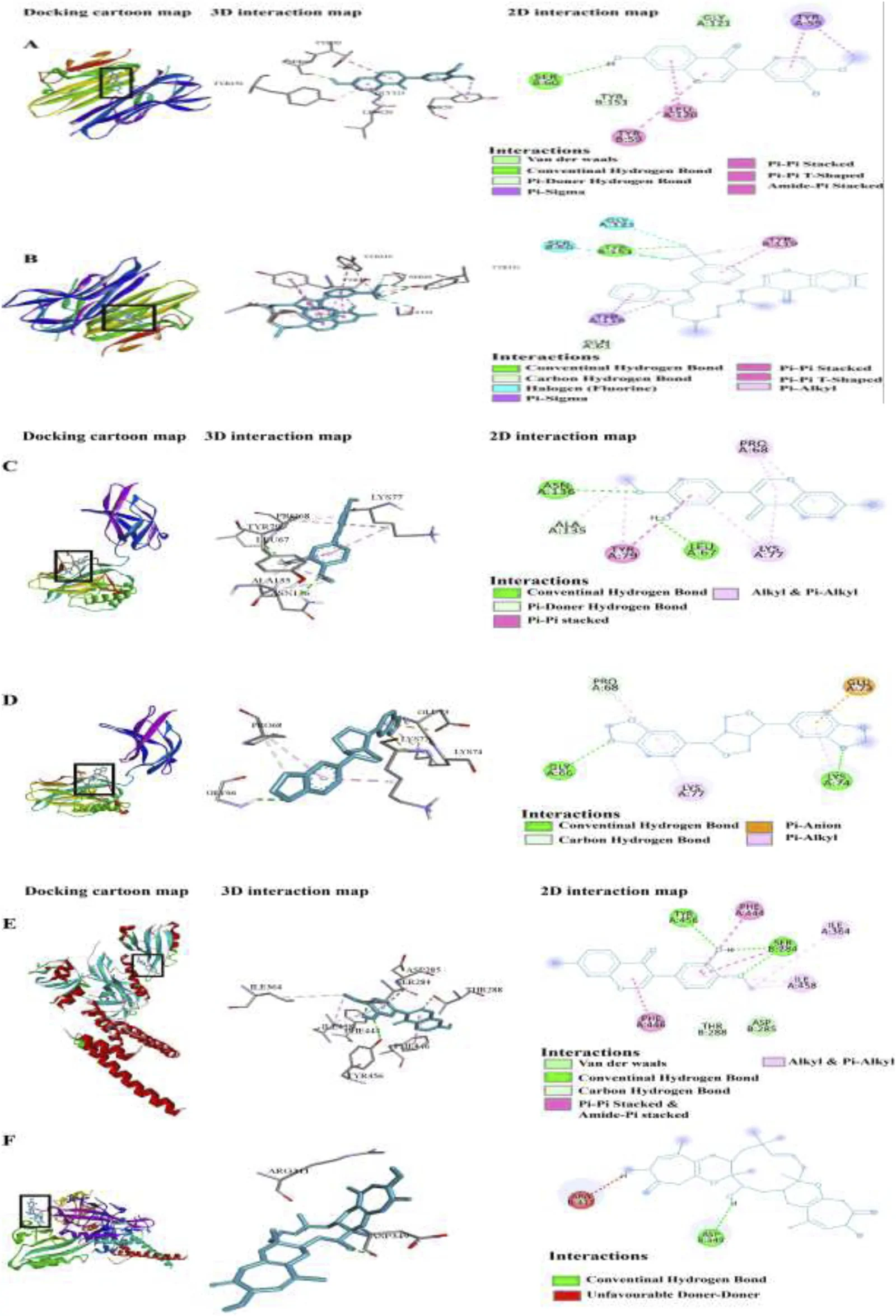
Docking results. **(A)** Calycosin + TNF-α; **(B)** SPD304 + TNF-α; **(C)** calycosin + NF-κB; **(D)** sesamin + NF-κB; **(E)** CALY-HIF-1α; **(F)** pycnidione-HIF-1α. Ligands are represented in cyan carbons.

This suggests that TNF-α, NF-κB, and HIF-1α may represent potential direct targets for calycosin to manage liver fibrosis. Docking results indicate the interaction mode among calycosin, and the three targets are based on hydrogen bonds and hydrophobic interactions, as shown in Table 6 and Figure 4.

**TABLE 6:** Docking results with core targets.

Target–ligand complex	Docking scores (kcal/mol)	Hydrogen bonds	Hydrophobic interactions	Halogen
Calycosin–TNF-α	−7.6	SER B:60	GLY A:121, TYR A:59, LEU A:120 TYR B:151, and TYR B:59	-
SPD304–TNF-α	−8.1	TYR B:151	TYR A:119, GLN A:61, and TYR B:119	GLY A:121 and SER B:60
Calycosin–NF-κB	−7	ASN A:136 and LEU A:67	ALA A:135, PRO A:68, TYR A:79, and LYS A:77	-
Sesamin–NF-κB	−7.7	GLY A:66 and LYS A:74	PRO A:68, LYS A:77, and GLU A:73	-
Calycosin–HIF-1α	−7.6	TYR A:456 and SER B: 284	PHE A:444, PHE A:446, ILE A:364, ILE A:458, ASP B:285, and THR B:288	-
Pycnidione–HIF-1α	−9.1	ASP B:349	ARG B:311 (unfavorable)	-

In each binding mode, the blue-scaled ball-and-stick models represent the ligand molecules, while the target was manifested in CPK and cartoon structure to demonstrate the binding sites in each target. The interactions were visualized as dashed lines, where bright green lines indicate hydrogen bonds, purple and light green colors represent hydrophobic interactions, and cyan color represents halogen interactions. Furthermore, the labels in the modes represent the amino acid residues of the targets.

### 3.3 The effect of calycosin on the *in vivo* EILI model

#### 3.3.1 The liver enzyme activities

**FIGURE 5:**
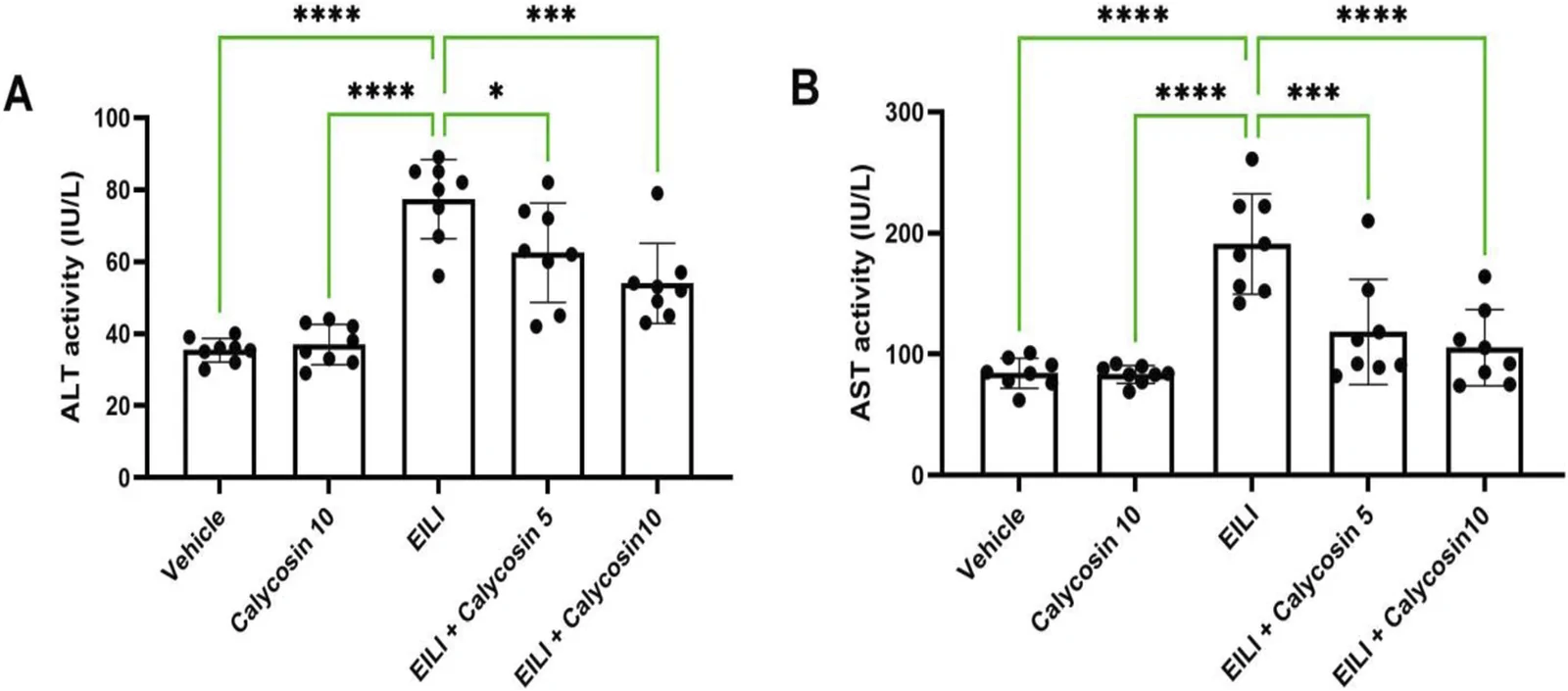
Serum liver enzyme activities. **(A)** Serum ALT activity and **(B)** serum AST activity. *, ***, and ****: significantly different at p < 0.05, p < 0.001, and p < 0.0001, respectively.

Figure 5 demonstrates the liver enzyme activities in the study groups, where significant differences were recorded among the study groups, as demonstrated by ANOVA: F (4,35) = 16.24 and R^2^ = 0.65 for AST; F (4,35) = 26.14 and R^2^ = 0.75 for ALT, p < 0.0001. Significant elevations were noticed in the EILI group compared to the vehicle group, whereas the EILI + calycosin (5 or 10 mg/kg) groups showed reduced values of AST and ALT.

#### 3.3.2 The ELISA assays for the liver NF-κB, TNF-α, and HIF-1α

Differences were significant among the study groups, as demonstrated by ANOVA: F (4,35) = 23.26 and R^2^ = 0.73 for NF-κB (p < 0.0001); F (4,35) = 26.93 and R^2^ = 0.755 for TNF-α (p < 0.0001); F (4,35) = 41.99 R^2^ = 0.82 for HIF-1α, p < 0.0001.

**FIGURE 6:**
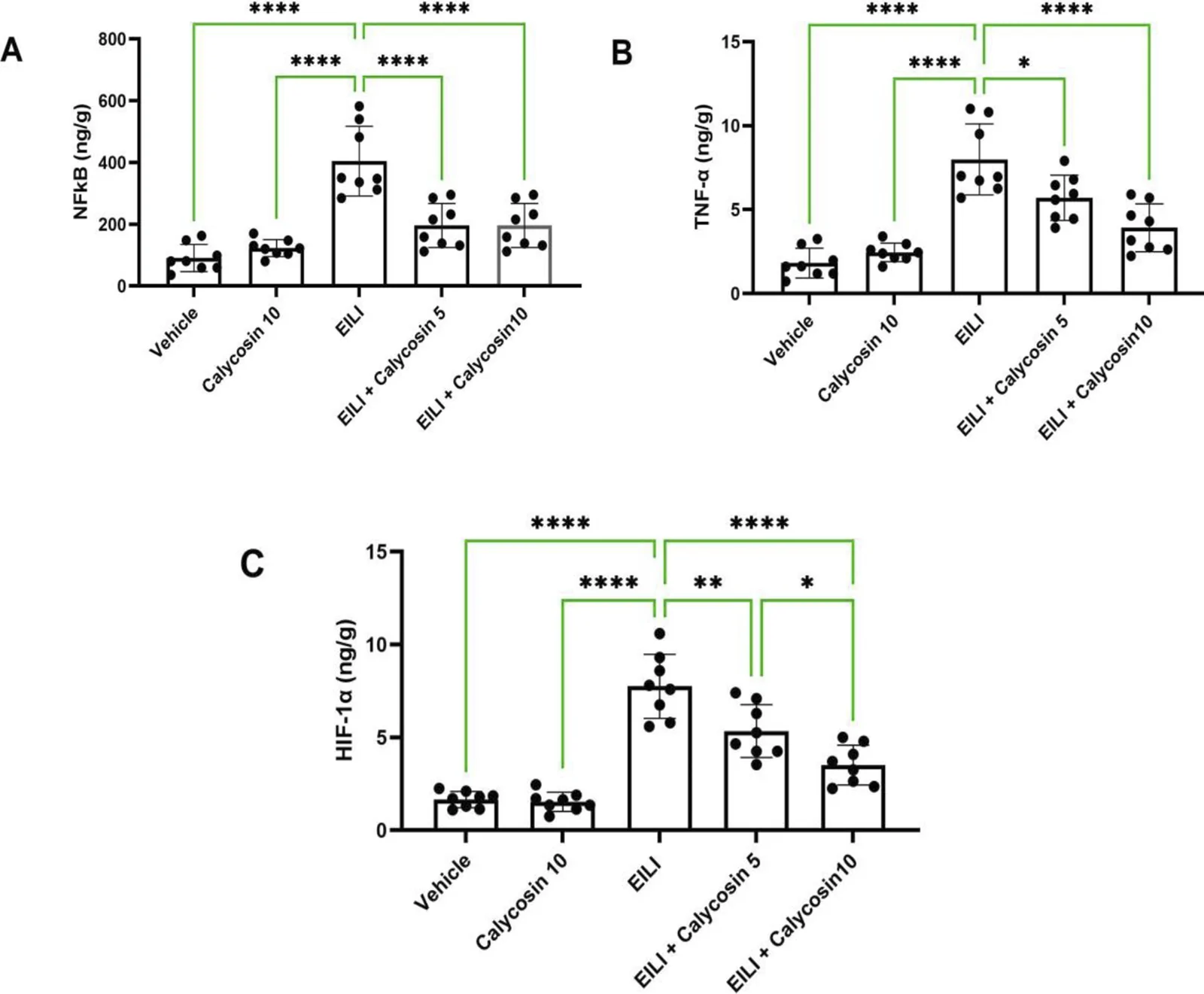
The levels of NF-κB, TNF-α, and HIF-1α in the liver homogenates. **(A)** Liver NF-κB, **(B)** liver TNF-α, and **(C)** HIF-1α. *, **, and ****: significantly different at p < 0.05, p < 0.01, and p < 0.0001, respectively.

The present results indicated that the calycosin (10 mg/kg) per se group was not significantly different compared to the vehicle group. Elevated levels of NF-κB, TNF-α and HIF-1α were observed in the EILI control group (Figures 6A–C). The EILI + calycosin (5 or 10 mg/kg) groups showed significant reductions in values of NF-κB and TNF-α compared to the EILI group, without a dose-dependent effect (Figures 6A,B). However, calycosin produced dose-dependent decreases in hepatic HIF-1α compared to the EILI group (Figure 6C).

#### 3.3.3 Hematoxylin and eosin staining

Liver sections from the double vehicle rats demonstrated normal histological architecture. The tissue specimen composition appeared in hexagonal classic hepatic lobules with ill-defined borders and central veins making the central axis. The portal area angles are formed of connective tissue stroma and portal triads. The portal tract comprised a hepatic artery terminal branch, a portal vein small branch, and a bile ductule.

**FIGURE 7:**
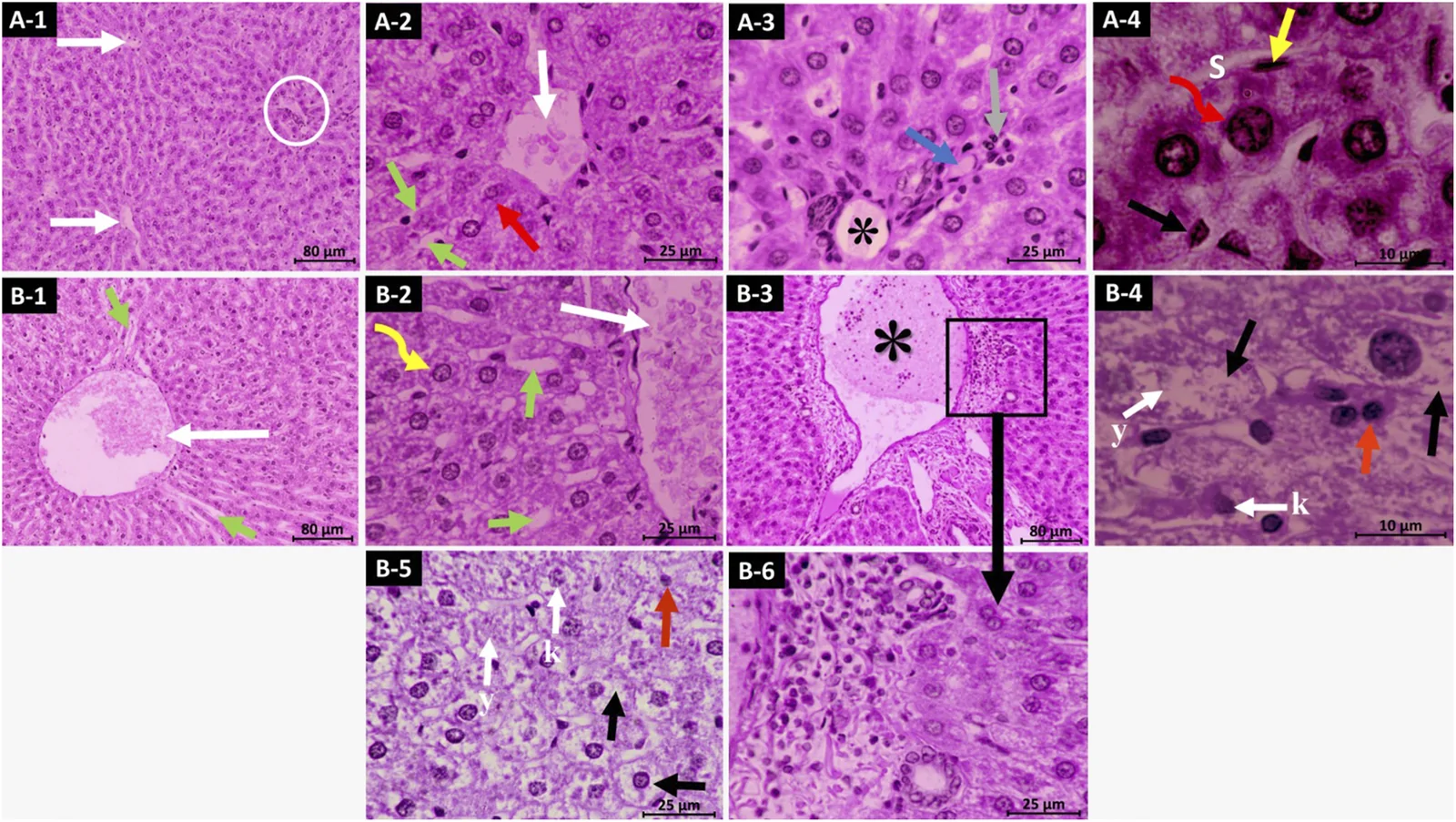
Photomicrograph of the liver section from experimental groups stained with hematoxylin and eosin. The vehicle group **(A1–A4)** showed hexagonal classic hepatic lobules having ill-defined borders and central veins creating the central axis (white arrows) and portal tract at their angles (white circle). The portal tract showing a portal venule (indicated by the star), hepatic arterioles (the blue arrow), and bile ductules (the grey arrows). Blood sinusoids (green arrows) were observed within the hepatocyte cords (red arrows), and the endothelial lining was flat (yellow arrow) and contained Kupffer cells (black arrow). Hepatocytes with polyhedral shape and large and rounded vesicular nuclei (curved red arrow) were also observed. **(B1–B6)** The EILI group showed disorganization in the liver architecture such as central vein dilatation and congestion (white arrow) and dilated blood sinusoids (green arrow). Congested dilated portal vein (*) and marked portal tract mononuclear cellular infiltrates were also observed (black square). **(B6)** Most of the hepatocytes showed cloudy swelling (curved yellow arrow) and hydropic degeneration such as vacuolated cytoplasm (black arrow) associated with pyknosis (red arrow), karyorrhexis (k), or karyolysis (y). (H and E: A1, B1, and B3 ×100), (H and E: A2, A3, B2, B5, and B6 ×400), and (H and E: A4 and B4 ×1000).

The classic hepatic lobule consisted of hepatocytes radiating from the central veins toward the periphery, where they were arranged in anastomosing cords. The hepatocytes appeared polygonal, containing a central rounded vesicular nucleus with normally dispersed chromatin and prominent nucleoli. Furthermore, binucleated hepatocytes were also observed. An acidophilic cytoplasm containing scattered basophilic granules was observed in the hepatocytes. In this group, 96.5% hepatocytes showed no affection, and the remaining (3.5%) showed mild affection. A network of blood sinusoids appeared within the hepatocyte plates, directed to the central vein. Blood sinusoids have lining by the endothelial cells and Kupffer cells. The endothelial cells formed a flat discontinuous layer with dark flattened nuclei, while Kupffer cells were distributed among the endothelial cells and appeared as large cells with rounded nuclei (Figures 7A1–A4).

Liver sections from the calycosin (10 mg/kg) per se group showed liver tissue with normal histological structure, similar to the control group.

The EILI control group showed many histological changes represented by marked disorganization in the hepatic tissue architecture, dilated and congested portal veins, markedly dilated central veins, and blood sinusoids. Marked mononuclear cellular infiltrates were observed surrounding the portal tract. Most of the hepatocytes showed degenerations in the form of cloudy swelling, which consists of swollen cells having pale cytoplasms, vacuolated cytoplasm (hydropic degenerating changes), and necrosis (nuclear change with cytoplasmic affection). Nuclear alterations were represented as nuclear pyknotic changes (darkly stained nuclei), karyolysis (lysed nuclei), and karyorrhexis (fragmented nuclei) (Figures 7B1–B6).

**FIGURE 8:**
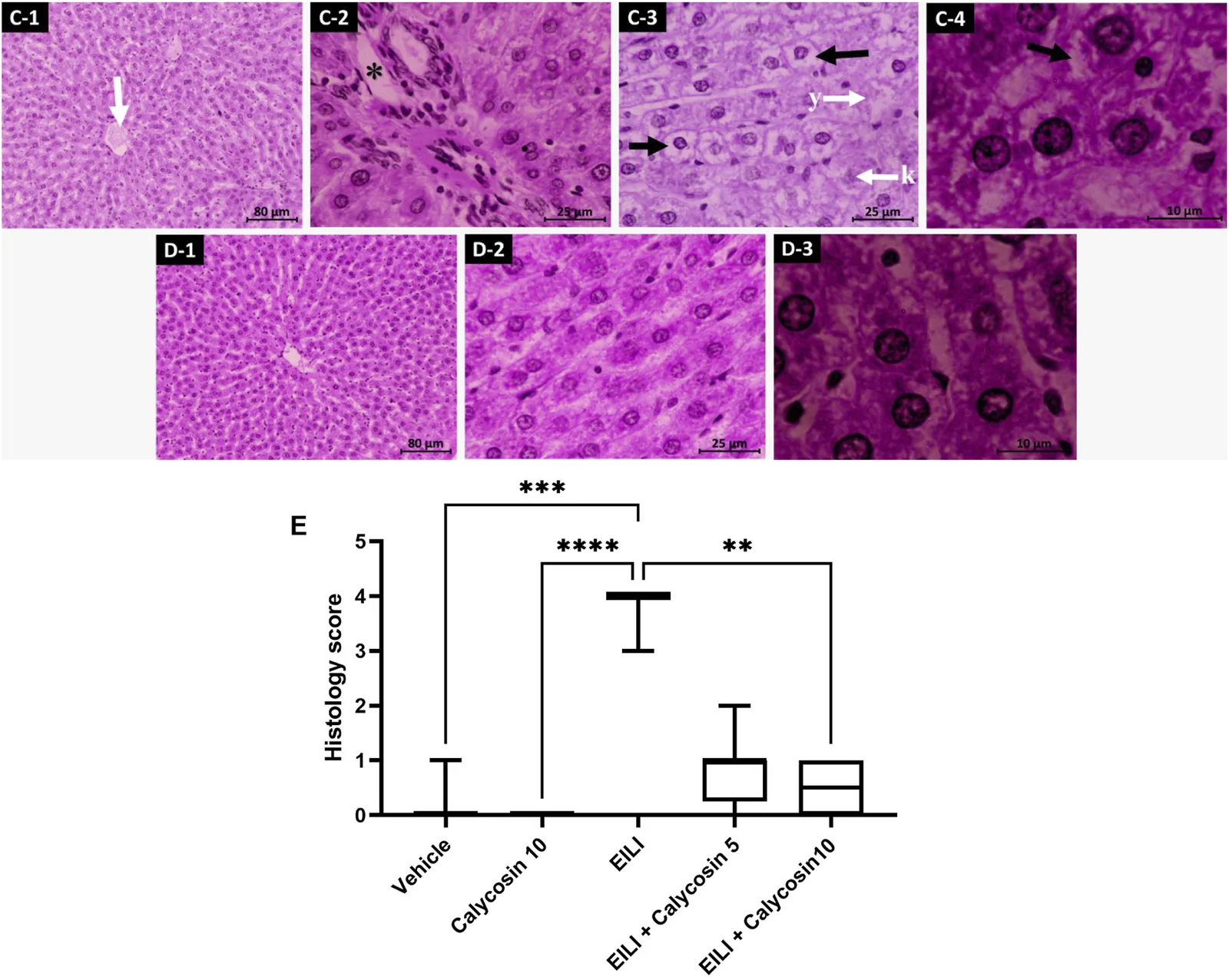
Photomicrograph of the liver section from experimental groups stained with hematoxylin and eosin. The EILI + calycosin (5 mg/kg) group **(C1–C4)** showed nearly normal liver architecture, with mild dilatation and congestion in the central vein (white arrow). Mildly congested and dilated portal vein (*) were also observed associated with scanty mononuclear cellular infiltration. Most hepatocytes appeared normal, and few hepatocytes contained cytoplasmic vacuoles (black arrows) and karyorrhetic (k) or karyolytic (y) nuclei. The EILI + calycosin (10 mg/kg) group **(D1–D3)** showed that hepatocytes and blood sinusoids were similar in structure to those in the control group (H and E: C1 and D1 ×100), (H and E: C2, C3, and D2 ×400), and (H and **(E)**: C4 and D3 ×1000). E) Column charts for the histology scoring in the study groups. ** and ***: significantly different at p < 0.01 and p < 0.001, respectively.

Liver sections from the EILI + calycosin (5 mg/kg) group revealed approximately normal liver architecture, but with mildly dilated and congested central veins. Mild congestion and dilation in the portal vein were also associated with scanty mononuclear cellular infiltration. Most hepatocytes appear normal, and a few contain cytoplasmic vacuoles and karyorrhetic or karyolytic nuclei (Figures 8C1–C4). Liver sections from the EILI + calycosin (10 mg/kg) group shows marked improvement in the hepatic architecture; there was no congestion or dilatation in the central veins and portal tracts. Hepatocytes and blood sinusoids appear similar in structure to the control group (Figures 8D1–D3). Figure 8E shows the histologic score in the EILI study groups; the score reported in the EILI + calycosin (10 mg/kg) group was lower than the score in the EILI group (p < 0.01).

#### 3.3.4 Masson’s trichrome staining

**FIGURE 9:**
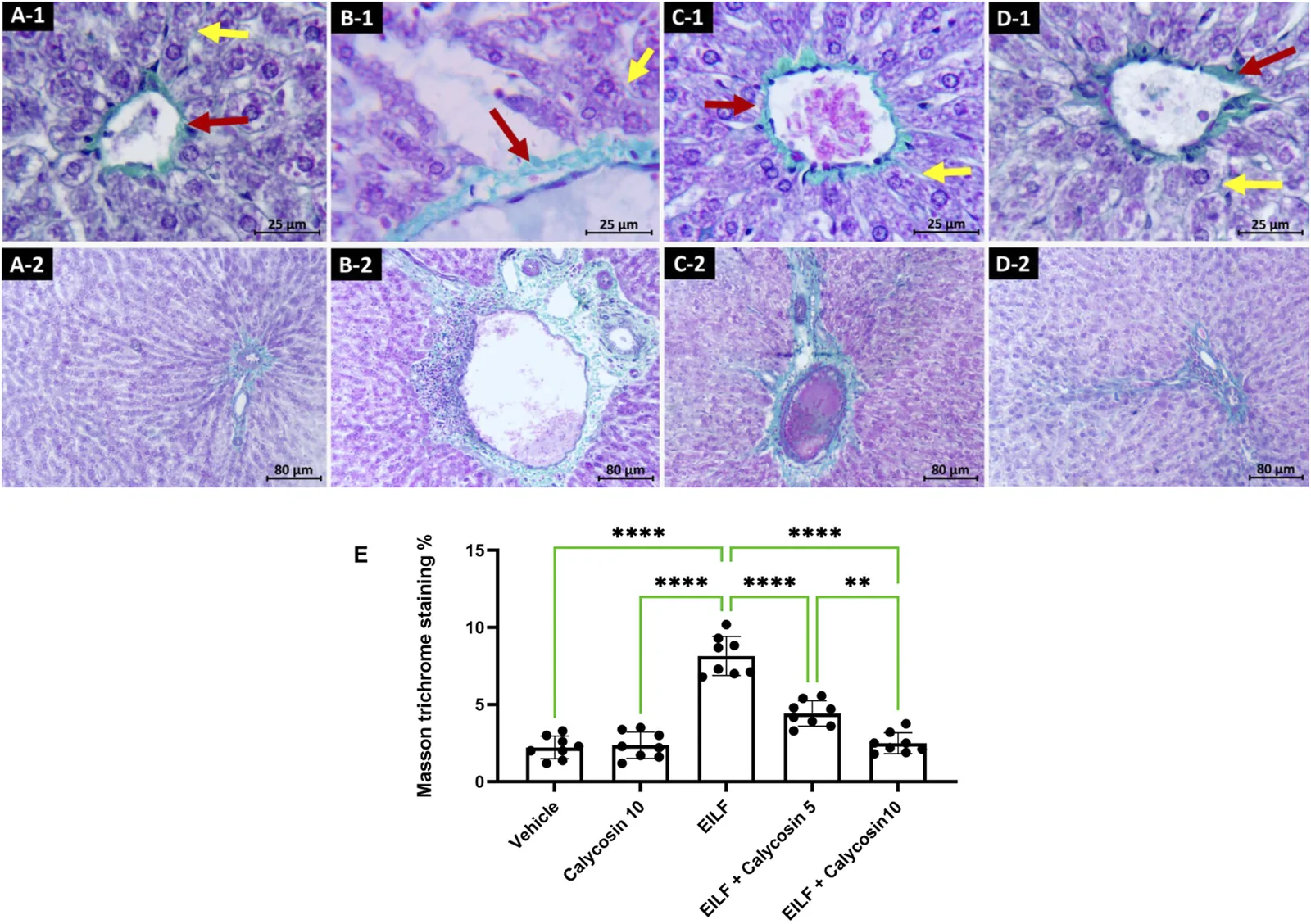
Liver section from different experimental groups stained with Masson’s trichrome staining. The vehicle group **(A1)** showed few collagen fibers surrounding the central vein (red arrow) and between blood sinusoids (yellow arrow). **(A2)** A few amounts of collagen fibers surrounding and between the portal tract were also observed. EILI group: **(B1)** showing mild collagen fiber content surrounding the central veins (red arrow) and minimal amount between blood sinusoids (yellow arrow). **(B2)** A markedly high collagen content surrounding and between portal tracts were also observed. EILI + calycosin (5 mg/kg) group: **(C1)** showing mild amount of collagen surrounding the central vein (red arrow) and blood sinusoids (yellow arrow). **(C2)** A moderate content of collagen fibers surrounding and within the portal tracts were also observed. **(D1,D2)** The EILI + calycosin (10 mg/kg) group showing minimal amount and distribution of collagen surrounding the central veins (red arrow), blood sinusoids (yellow arrow), and portal tracts similar to the vehicle group (Masson’s trichrome: A1, B1, C1, and D1 ×400) and (Masson’s trichrome: A2, B2, C2, and D2 ×1000). **(E)** Column charts for the area of Masson’s trichrome staining. ** and ***: significantly different at p < 0.01 and p < 0.001, respectively.

As shown in Figures 9A1,A2, Masson’s trichrome staining for sections from the double vehicle group demonstrated a scant amount of collagen fibers surrounding the central vein and blood sinusoids. A few amounts of collagen fibers appeared between and surrounding the portal tract. The area % of collagen fibers was 2.22% ± 0.73%. The same results were found in the calycosin per se group. The collagen area % of was 2.38 ± 0.86; there was no significant difference (p ≥ 0.05) compared to the vehicle group. In contrast, the EILI group revealed an increased content of collagen fibers around central veins. Furthermore, a markedly increased collagen fiber content was observed surrounding and within portal tracts and the hepatocyte cords (Figured 9B1,B2). The area% of collagen fibers was 8.156% ± 1.26% (Figure 9E).

Moreover, the EILI + calycosin (5 mg/kg) group revealed a mild presence of collagen fibers surrounding the central veins and blood sinusoids. A moderate presence of collagen fibers was identified surrounding and between portal tracts (Figures 9C1,C2). The area % of collagen fibers was 4.43% ± 0.82% (Figure 9E); the area % of collagen was significantly lower than that in the EILI group. Furthermore, the EILI + calycosin (10 mg/kg) group showed the amount and distribution of collagen fibers surrounding blood sinusoids (yellow arrow), the central veins (red arrow), and portal tracts (Figures 9D1,D3) similar to some extent to the vehicle group. The area % of collagen fibers was 2.49 ± 0.67 (Figure 9E); the area of collagen deposition % was significantly lower than that in the EILI control group but not different (p < 0.05) from that in the EILI + calycosin (5 mg/kg) group.

It is important to highlight significant differences among the study groups, as demonstrated by ANOVA test: F (4,35) = 63.31 and R^2^ = 0.88 for the area of collagen deposition in Figure 9E, p < 0.0001.

#### 3.3.5 Immunohistochemistry examination of α-smooth muscle actin

**FIGURE 10:**
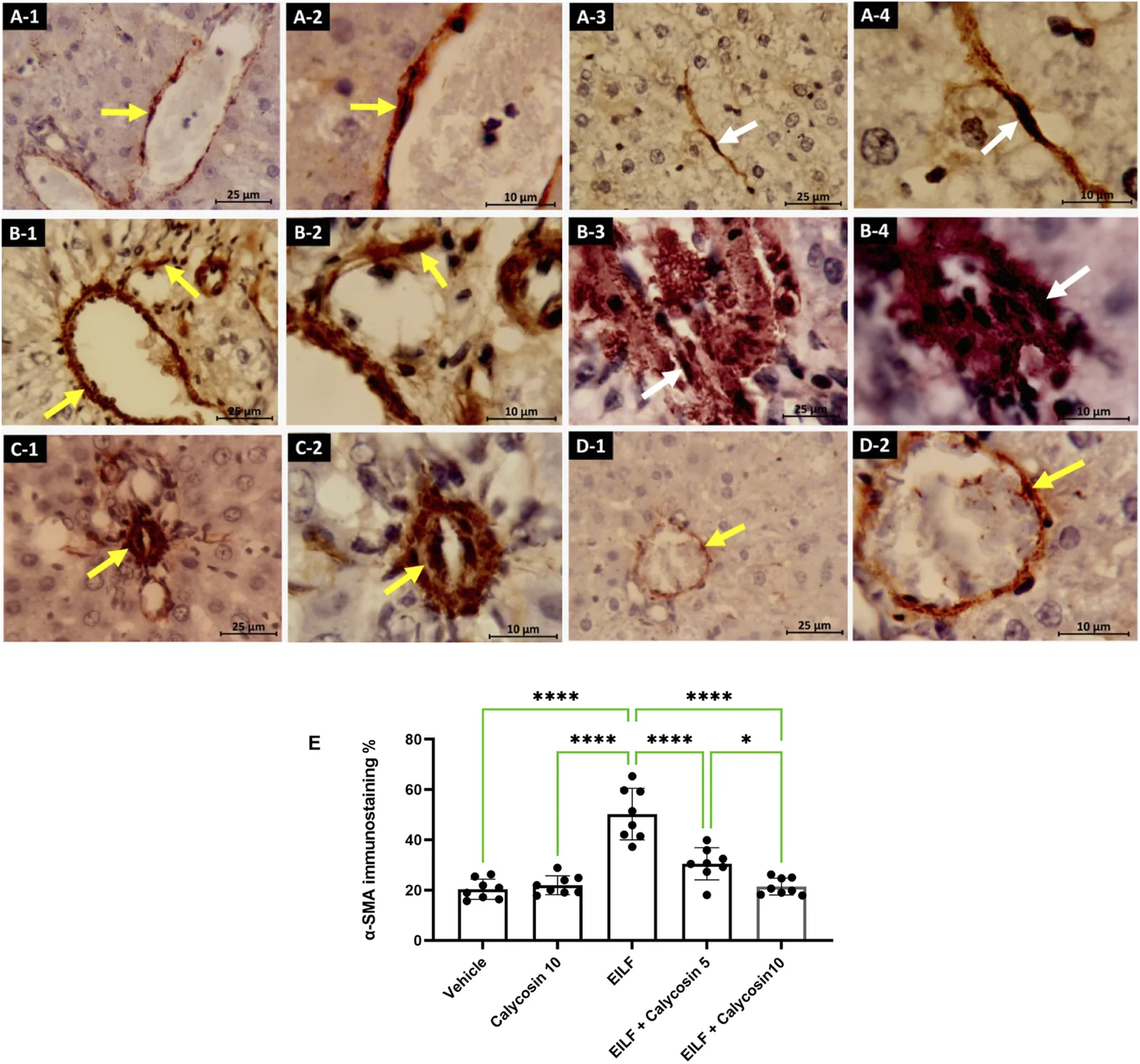
α-SMA protein immunostaining in the liver tissue section from different experimental groups. **(A1–A4)** The double vehicle group showing a small area of α-SMA-protein-positive HSC around the vascular wall of the portal tracts (yellow arrows) and between hepatocytes (white arrows). **(B1–B4)** The EILI control group showed a marked increase in the area of the α-SMA protein around the vascular wall of the portal tracts (yellow arrows), inflammatory cells, and between fibrous tissue (white arrows). **(C1,C2)** EILI + calycosin (5 mg/kg) group showing a mild decrease in the area % of α-SMA-protein-positive HSCs around the vascular wall of the portal tracts (yellow arrows) and inflammatory cells compared to the EILI group. **(D1,D2)** EILI + calycosin (10 mg/kg) group showing the low area% of α-SMA-positive-HSCs around the vascular wall of the portal tracts compared to the EILI group (α-SMA protein immunostaining: A1, A3, B1, B3, C1, and D1 ×400) and (α-SMA protein immunostaining: A2, A4, B2, B4, C2, and D2 ×1000). Column charts for the area of immunohistochemical staining. * and ****: significantly different at p < 0.05 and p < 0.0001, respectively.

Examination of the α-SMA-immunostained hepatic tissue from the double vehicle group showed very mild α-SMA staining around the vascular wall of the portal tracts and between hepatocytes (Figures 10A1–A4). The mean area of immunostaining of α-SMA-positive cell expression was 20.16% ± 4.16% (Figure 10E). The same immunohistochemical results were detected in the calycosin per se group regarding the distribution and area of α-SMA protein reaction. The area of α-SMA immunostaining was 21.98% ± 3.77% (Figure 10E).

Assessment of the α-SMA protein expression in the hepatic tissue of the EILI control group demonstrated a marked increase in the immunostaining of α-SMA around the vascular wall of the portal tracts, inflammatory cells, and between fibrous tissue (Figures 10B1–B4). The mean area of α-SMA immunostaining was 51.39.0% ± 10.19% (Figure 10E). There was a statistically significant increase when compared to the vehicle group.

Furthermore, α-SMA protein expression in the hepatic tissue of the EILI + calycosin (5 mg/kg) group showed mild immunostaining for α-SMA around the vascular wall of the portal tracts and inflammatory cells (Figures 10C1,C2). The mean area of α-SMA immunostaining was 30.51% ± 6.45% (Figure 10E). The results highlighted a significant decrease in α-SMA area % compared to that in the EILI rats.

Evaluation of α-SMA protein expression in the hepatic tissue of the EILI + calycosin (10 mg/kg) group revealed a low area of α-SMA immunostaining, around the vascular wall of the portal tracts, nearly similar to the vehicle group (Figure 10 D1 and D2). The mean area of α-SMA immunostaining was 21.42 ± 3.76 (Figure 10E). The results displayed a significant decrease in α-SMA area % compared to that in the EILI group and the EILI + calycosin 5 mg/kg group.

It is important to highlight significant differences among the study groups, as demonstrated by ANOVA: F (4,35) = 34.02 and R^2^ = 0.79 for the area of α-SMA immunostaining in Figure 10E, p < 0.0001.

#### 3.3.6 TEM examination

**FIGURE 11:**
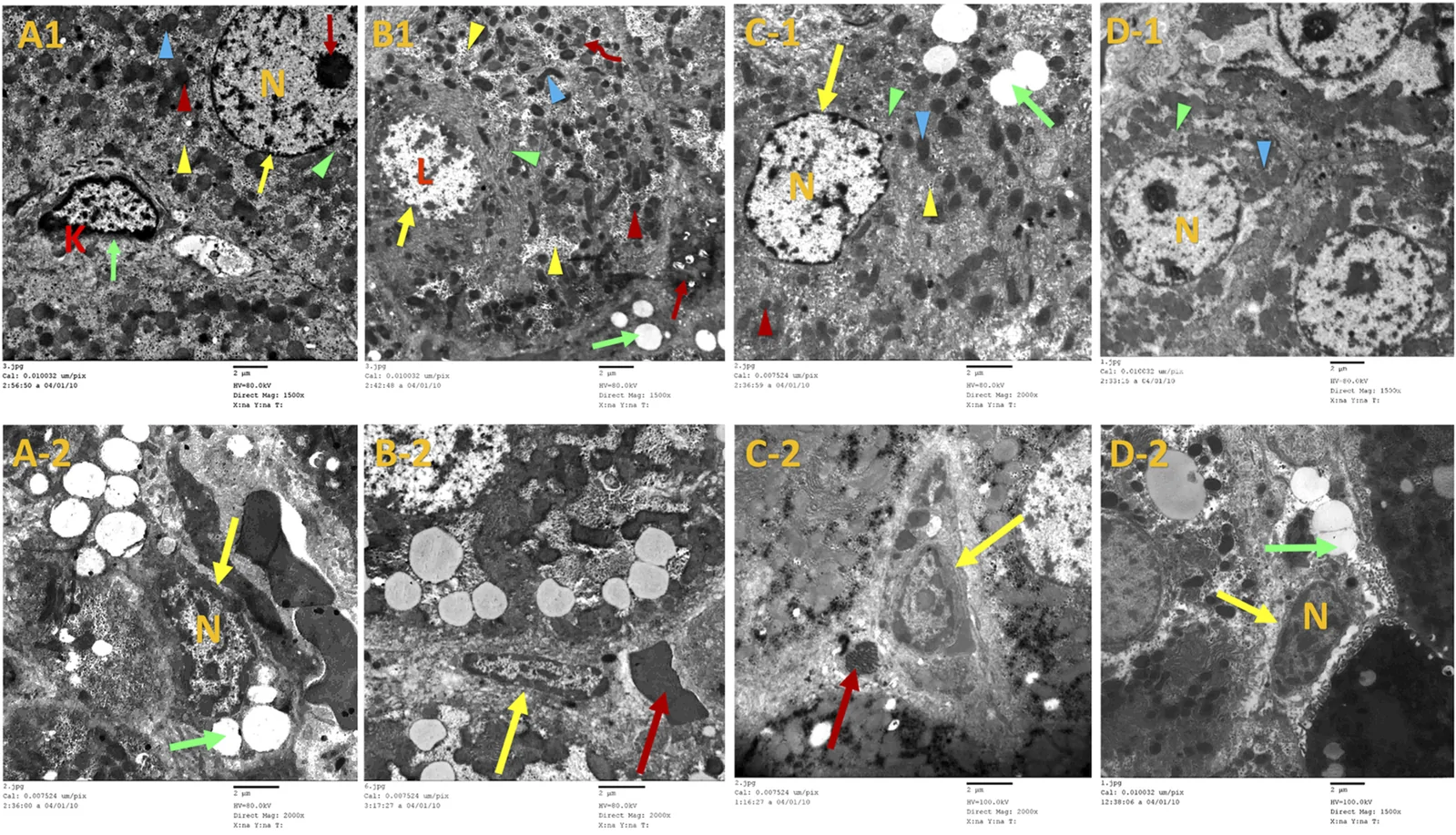
Electron micrograph of liver ultrathin sections from different experimental groups. The double vehicle group **(A1)** showing hepatocytes consisting of cytoplasm and nucleus. The cytoplasm contains plenty of rounded or oval-shaped mitochondria (blue arrowhead), rough endoplasmic reticulum (green arrowhead), peroxisomes (red arrowhead), and electron-dense glycogen granules (yellow arrowhead). The nucleus (N) is euchromatic, round in shape, containing prominent nucleoli (red arrow), and surrounded by a nuclear membrane (yellow arrow). Kupffer cells (K) with indented dark nuclei (green arrow) were also observed. **(A2)** HSC (yellow arrow) between the blood sinusoids, with a large irregular heterochromatic nucleus (N) and large fat droplets (green arrow). EILI control group **(B1)** showing multiple pleomorphic shapes of mitochondria (blue arrowhead), dilatation of rough endoplasmic reticulum cisternae (green arrowhead), numerous glycogen granules (yellow arrowhead), abundant peroxisomes (red arrowhead), and phagosomes (red curved arrow). Nuclear degenerative changes in the form of loss of nuclear membrane (yellow arrow) and lysis of nuclear chromatin and nucleoli (L). Aggregates of apoptotic hepatocyte remnants were observed between the hepatocytes. In addition, large fat droplets were observed between blood sinusoids (green arrow). **(B2)** HSC (yellow arrow) alteration to myofibroblast-like cells located between the blood sinusoids associated with loss of fat droplets and formation of collagen fibrils bundles (red arrow) within hepatocytes. The EILI + calycosin (5 mg/kg) group **(C1)** showing mitochondria partially normal in shape and matrix appearance (blue arrowhead), normal rough endoplasmic reticulum (green arrowhead), few amounts of glycogen granules (yellow arrowhead), and scanty peroxisomes (red arrowhead). Nucleus (N) revealing irregular shape and nuclear membrane (yellow arrow), associated with disorganization in their nucleoplasm and absence of nucleoli. A few large fat droplets (green arrow) were observed between the blood sinusoids. **(C2)** HSC (yellow arrow) still appearing as a myofibroblast-like cell, characterized by loss of fat droplets and formation of fine bundles of collagen fibrils (red arrow) between hepatocytes and the extracellular matrix, especially in the areas of the portal tract. The EILI + calycosin (10 mg/kg) group **(D1)** showing strong restoration of cellular components of hepatocytes and appeared close to the vehicle group ultrastructure, expressed by numerous mitochondria (blue arrowhead) with normal shape, plenty of rough endoplasmic reticulum (green arrowhead), and normal appearance of nucleus (N). **(D2)** HSC (yellow arrow) between blood sinusoids with large irregular heterochromatic nucleus (N) and large fat droplets (green arrow).

Examination of ultrathin sections from the double vehicle group showed normal hepatocytes with polyhedral shapes containing large rounded euchromatic nuclei with prominent nucleoli and surrounded by a nuclear membrane. The hepatocyte cytoplasm encompasses cell organelles, plenty of mitochondria that appreared round or oval shapes in close to the secondary lysosomes, endoplasmic reticulum, and glycogen. A Kupffer cell appeared in the lining of the blood sinusoids having dark nucleus. Glycogen appeared as scattering dense elements (Figure 11A1). Hepatic stellate cells (HSCs) were observed in the spaces of Disse between the hepatocytes and blood sinusoids, with their relatively large, irregular, heterochromatic nuclei. Large fat droplets were observed in their cytoplasm (Figure 11A2). Examining ultrathin sections of the livers from the calycosin per se group showed the same normal ultrastructure as that in the vehicle group.

Liver ultrathin sections from the EILI group revealed rarefaction of cytoplasmic organelles represented with multiple pleomorphic (multi abnormal shapes) mitochondria associated with an unusually electron-dense matrix and dilatation of rough endoplasmic reticulum cisternae. Moreover, numerous electron-dense black glycogen granules, abundant electron-dense peroxisomes, and phagosomes were also observed. Hepatocytes also showed nuclear degenerative changes in the form of loss of nuclear membrane and lysis of nuclear chromatin and nucleoli. Aggregates of apoptotic hepatocyte remnant bodies were found between the hepatocytes. In addition, large fat droplets were observed between blood sinusoids (Figure 11B1). HSCs between blood sinusoids showed alteration in cell morphology and transformation to a myofibroblast-like cell, as evidenced by loss of fat droplets and formation of collagen fibrils in bundles between hepatocytes and extracellular matrix, especially in areas of the portal tract due to HSC activities (Figure 11B2).

Examination of ultrathin sections of livers obtained from animals in the EILI + calycosin (5 mg/kg) group showed partial improvement of degenerative changes in the hepatocytes, as expressed by amelioration of the rarefaction of cytoplasmic organelles. Some mitochondria showed moderate improvement in their shape and matrix. Normal rough endoplasmic reticulum, few amounts of glycogen granules, and scanty peroxisomes were also observed. Hepatocytes showed irregular nuclei and nuclear membrane associated with disorganization in their nucleoplasm and absence of nucleoli. In addition, a few large fat droplets were observed between the blood sinusoids (Figure 11C1). HSCs showed a mild decrease in their activity but still appeared as a myofibroblast-like cell, characterized by loss of fat droplets and formation of fine bundles of collagen fibrils between hepatocytes and extracellular matrix, especially in areas of the portal tract (Figure 11C2).

Examination of ultrathin sections from rats in the EILI + calycosin (10 mg/kg) group showed great restoration of cellular components of hepatocytes and appeared nearly close to the vehicle group ultrastructure, characterized by numerous mitochondria with normal shape, plenty of rough endoplasmic reticulum, and normal nuclear appearance (Figure 11D1). HSCs were observed between blood sinusoids, with normal appearance in the form of large irregular heterochromatic nuclei and large fat droplets (Figure 11D2).

## 4 Discussion

The present study is an integrative *in silico*–*in vivo* experimental pharmacology study incorporating network pharmacology analysis, molecular docking, and a controlled *in vivo* rat model. The animal model (Wistar rat, n = 8) included serum biochemical, ELISA, histological (H&E and Masson’s trichrome), IHC (α-SMA), and transmission electron microscopic endpoints. This study utilized a multi-staining approach to evaluate the hepatoprotective potential of calycosin against EILI. By integrating H&E for structural morphology, Masson’s trichrome for assessment of fibrosis, and α-SMA immunohistochemistry for molecular signaling, we established a clear link between cellular damage, the fibrogenic response, and dose-dependent therapeutic recovery.

With H&E staining, the control groups (I and II) exhibited the classic hallmarks of healthy hepatic architecture. The presence of well-defined hexagonal lobules, radiating cords of hepatocytes, and a preserved portal triad (comprising the portal venule, hepatic arteriole, and bile ductule) confirms the baseline integrity of the hepatic tissues in the EILI control group.

In contrast, the EILI group displayed profound structural disturbances characteristic of chronic hepatic injury such as marked dilatation and congestion of the central and portal veins, suggesting a disruption in hepatic blood flow and increased sinusoidal pressure. Cloudy swelling and hydropic degeneration were also observed. Furthermore, the presence of pyknosis, karyorrhexis, and karyolysis confirms irreversible cellular damage and programmed cell death (necrosis/apoptosis) resulting from the pathogenic insult. These findings are consistent with previous studies suggesting that ethanol induces oxidative stress, resulting in the breakdown of hepatic cord organization and microvascular congestion (Gohari Mahmoudabad et al., 2023).

The transition from the pathology group to the treated groups revealed a significant, dose-dependent improvement in hepatic morphology. In the low-dose group, while these sections showed a trend toward recovery, the persistence of “scanty mononuclear cellular infiltration” and localized cytoplasmic vacuolation suggests that the lower dose of calycosin can initiate repair and partly resolve the inflammatory response. However, high-dose intervention effectively restored the liver architecture to a state nearly indistinguishable from the control groups. The absence of congestion and the normalization of the blood sinusoids suggest that the treatment at this concentration possesses potent cytoprotective properties, likely through the stabilization of hepatocyte membranes and the reduction in oxidative stress. These results are consistent with the results obtained by Zhang et al. (2021).

The serum ALT and AST are two classic biochemical parameters of liver injury. Under physiological conditions, hepatocytes contain normal levels of ALT and AST, but when the hepatocytes are damaged, high amounts of the enzymes release to the blood stream, leading to increases in the serum levels of ALT and AST (Arumugam et al., 2022). In the present study, the EILI group showed significantly increased ALT and AST levels. Importantly, calycosin was able to significantly reduce serum enzyme activities. Hence, this was a good indicator to evaluate calycosin and to conclude about its ability to improve the liver function, along with liver integrity and alleviation of EILI.

Furthermore, the targeted markers of inflammation, such as TNF-α, NF-κB, and HIF-1α, were elevated in the EILI group but downregulated upon treatment with calycosin. However, there was dose–response inconsistency in the effect of calycosin on these markers. The results do not show clear dose dependence. Indeed, calycosin showed an dose-dependent reducing effect on HIF-1α, but not in case of TNF-α and NF-κB. This may be explained by the saturation effect and biological variability among the targeted markers.

MT staining was used to specifically highlight collagen deposition. In the vehicle group, minimal fibers were detected surrounding the central veins and within the portal tracts, maintaining the delicate balance of the hepatic architecture. However, the EILI group exhibited a significant shift toward fibrogenesis. The “marked increase” in collagen fibers, particularly between the cords of hepatocytes and around the portal tracts, indicates the activation of HSCs; these results are consistent with those of Alahmari et al. (2022) and Mittal et al. (2025). This transformation from a quiescent state to an active, myofibroblast-like phenotype is the primary driver of the fibrotic response observed in our pathological model. The statistically significant increase in mean area % of immunostaining quantitatively confirms this transition from simple inflammation to structural remodeling.

Calycosin treatment demonstrated a dose-dependent ability to mitigate collagen deposition (Zhang et al., 2021). Although there was a slight reduction in the density of fibers in the low-dose group compared to that in the EILI group, the results remained “statistically significant” (p < 0.05). The presence of moderate collagen around portal tracts suggests that at lower concentrations, the treatment may slow, but not entirely halt, the fibrotic cascade. Most notably, the calycosin high-dose group effectively arrested the fibrotic process. The amount and distribution of collagen were restored to levels similar to the vehicle control group, which suggests a near-complete resolution of the fibrotic changes. This highlights the high dose’s potential as a potent anti-fibrotic agent, capable of preventing the progression of liver injury into more permanent stages of cirrhosis. This can be explained by the reduction in the production of collagen I to mitigate EILI (Wang et al., 2019; Zhang et al., 2021).

To elucidate the cellular mechanisms underlying the observed structural and fibrotic changes, we performed immunohistochemical staining for α-SMA. This protein serves as a specific marker for the activation of HSCs. In a healthy liver, HSCs remain in a quiescent, vitamin A-storing state; however, upon injury, they transmigrate into myofibroblast-like cells that express α-SMA and synthesize the bulk of the extracellular matrix. In the vehicle and calycosin 10 mg/kg per se groups, α-SMA expression was minimal and largely restricted to the smooth muscle cells within the vascular walls of the portal tracts. This represents the physiological baseline of the liver. However, the EILI group exhibited a “marked increase” in the α-SMA area %. This expression was not limited to vascular structures but extended into the parenchyma, specifically around inflammatory cells and within fibrous bands. The significant increase in the area of α-SMA % confirms that HSC activation is a primary driver of the architectural disorganization and fibrosis observed in this model, which is consistent with previous results from a different laboratory (Mittal et al., 2025).

In a previous study, calycosin showed a potent inhibitory effect on the activation of these fibrogenic cells (Zhang et al., 2021). In the present EILI model, calycosin produced a dose-dependent significant decrease in α-SMA production that was qualitatively observed around the portal tracts and inflammatory zones. This suggests that even at a lower concentration, the treatment effectively blunts the signal for HSC activation, preventing the widespread myofibroblastic transformation observed in the EILI group.

The results for the calycosin high-dose group were even more definitive. The density and distribution of α-SMA-positive cells were nearly identical to the control group. This indicates that the high-dose treatment successfully maintains HSCs in their quiescent state or prevents their activation entirely, thereby preserving the liver from fibrotic remodeling. This result was consistent with previous studies, where it was found that calycosin upregulates estrogen receptor β expression and inhibits STAT3 phosphorylation to hinder hepatic stellate cell activation and collagen deposition (Wang et al., 2022). In agreement, two previous experimental reports highlighted an anti-fibrotic effect for calycosin (Guo et al., 2018; Zhang et al., 2021).

Duan et al. (2017) reported that calycosin reduced the accumulation of TGs and liver fibrosis in mice with non-alcoholic steatohepatitis. The mechanism was linked to the inhibition of TG synthesis, stimulation of fatty acid β-oxidation, and inhibition of HSC activation (. In another study conducted in mice fed on a high-fat diet, calycosin was reported to prevent the changes in liver histopathology and lipid accumulation; these favorable effects were mediated mainly via the increase in glycogen synthase kinase-3 beta (Duan et al., 2018). Calycosin has been shown to attenuate acute liver injury induced by CCl4 in C57BL/6 mice with promotion of hepatocyte mitosis and activation of FXR and STAT3 (Chen et al., 2015). The authors used serum liver enzyme activities, liver histopathology, and TUNEL assays for supporting this protective effect.

When the findings from H&E staining (cellular integrity), Masson’s trichrome staining (collagen deposition), and α-SMA immunohistochemistry (HSC activation) are considered together, a clear therapeutic profile emerges. The injury leads to hepatocyte necrosis (H&E), which triggers an inflammatory response and the subsequent activation of HSCs (α-SMA). These activated cells then deposit excessive collagen (Masson’s trichrome stain), leading to the observed architectural disorganization. Calycosin treatment appears to intervene at multiple levels. By reducing cellular damage and inflammation, it subsequently prevents the activation of HSCs (as evidenced by low α-SMA expression); this ultimately results in the negligible collagen deposition and restored architecture observed in the EILI + calycosin high-dose group. The ability of the high-dose calycosin treatment to return α-SMA levels to baseline suggests that it possesses strong anti-inflammatory and anti-fibrotic properties, making it a promising candidate for preventing the progression of hepatic injury.

Limitations of this study also include the following: 1) using the lowest-possible probability threshold in target prediction and the moderate docking affinity showed by calycosin to the target proteins, 2) the absence of *in vitro* mechanistic validation for the target pathway in hepatic stellate cells or hepatocytes, 3) small sample size, 4) lack of a reference hepatoprotective drug and long-term assessment, 5) absence of pharmacokinetics/toxicity data, and 5) relying solely on ELISA protein measurement without more mechanistic validation.

Another limitation of the present study is that only male animals were included. Sex is increasingly recognized as an important biological variable in EILI and liver fibrosis. Previous studies have demonstrated that female animals may exhibit greater susceptibility to EILI (Thurman, 2000; Eagon, 2010) due to differences in ethanol metabolism, hormonal regulation, gut permeability, and inflammatory responses (Bizzaro et al., 2023). For example, estrogen has been reported to enhance Kupffer cell activation and increase responsiveness to lipopolysaccharide (Ikejima et al., 1998), potentially leading to greater hepatic inflammation and fibrogenic signaling following EILI. Conversely, sex hormones may also influence oxidative stress pathways, lipid metabolism, and hepatic stellate cell activation, resulting in differences in fibrosis progression between male and female animals.

The magnitude of liver injury, inflammatory responses, and fibrotic changes observed in this study may differ in female animals. We now explicitly state that the findings of the present study do not fully translate to female animals, and future studies should evaluate both sexes to determine the generalizability of the observed effects.

## 5 Conclusion

The present results demonstrated that calycosin attenuated EILI in rats, prevented the increase in the liver enzymes, and attenuated the histopathological/ultrastructural manifestations and collagen accumulation in the liver. Hence, the protective and healing properties of calycosin are to be investigated. It will be meaningful to develop calycosin as a new natural medicine for treatment of liver diseases.

The strength of this study is that it is a multi-level mechanistic convergence. The study integrates three independent lines of evidence—network pharmacology target identification, molecular docking to explore possible interaction with quantitative predicted binding scores against thee hub proteins (TNF-α, NF-κB, and HIF-1α), and *in vivo* ELISA quantification of the same three targets in liver homogenates, although causality has not been established. This created a self-reinforcing mechanistic argument that is stronger than any single-method approach.

Another strength point is using multi-stain histological characterization. The use of three complementary tissue assessment methods—H&E for architectural morphology, Masson’s trichrome for quantitative collagen optical density, and α-SMA immunohistochemistry. For HSC activation, α-SMA immunohistochemistry applied to the same tissue blocks provided a rigorous, multi-dimensional characterization of the fibrotic phenotype and its treatment-induced reversal. This is a dose–response design with dose differentiation. The inclusion of two calycosin doses (5 and 10 mg/kg) produces a dose–response gradient confirmed across multiple endpoints (ALT/AST, collagen density, α-SMA expression, and TEM ultrastructure), leading to internal validity to the hepatoprotective conclusion beyond what a single-dose study could support.

## Data Availability

The raw data supporting the conclusions of this article will be made available by the authors, without undue reservation.
