# Supplementary material for: Calycosin protects from histopathological and ultrastructural changes in ethanol-induced liver injury and exhibits predicted binding affinity to TNF-α/NF-κB/HIF-1α proteins
Source: Front Pharmacol. 2026 Aug 6;17:1860075. doi: 10.3389/fphar.2026.1860075 (PMC13489801; doi:10.3389/fphar.2026.1860075)
Supplement: Supplementary file 1 [file Supplementaryfile1.pdf]

Supplementary file 2

UniProt verified rat orthologs of the key human targets

Human gene/protein	Human UniProt ID	Rat ortholog	Rat UniProt ID	Match count
**Tumor necrosis factor (TNF)**	**P01375**	**Tumor necrosis factor (TNF)**	**P16599**	10/10
**Nuclear factor NF-kappa-B p105 subunit (NFKB1)**	**P19838**	**Nuclear factor NF-kappa-B p105 subunit (NFKB1)**	**Q63369**	8/10
**Hypoxia-inducible factor 1-alpha (HIF1A)**	**Q16665**	**Hypoxia-inducible factor 1-alpha (HIF1A)**	**O35800**	10/10
