# Supplementary material for: Calycosin protects from histopathological and ultrastructural changes in ethanol-induced liver injury and exhibits predicted binding affinity to TNF-α/NF-κB/HIF-1α proteins
Source: Front Pharmacol. 2026 Aug 6;17:1860075. doi: 10.3389/fphar.2026.1860075 (PMC13489801; doi:10.3389/fphar.2026.1860075)
Supplement: Supplementary file 2 [file Supplementaryfile2.pdf]

Supplementary file 2

Reperforming network pharmacology under stricter selection criteria

Methods

GeneCards Targets with relevance score more than 6, SuperPred targets with probability more than 50%, and Swiss target prediction targets with degree more than 0.09 were considered for intersection analysis.

Results

Intersection analysis yielded 33 common targets with EGFR and HIF1A being the top hub targets with degree value equal to 6 for both, under confidence level of 0.7.

Table 1.

Intersected targets with their relevant degree value

Target	degree
EGFR	6
HIF1A	6
CREBBP	3
NFE2L2	3
PLAU	3
CDK1	2
IL2	2
PLAT	2
PPARA	2
SERPINE1	2
AXL	1
CTSD	1
GPBAR1	1
GSK3B	1
LGALS3	1
NR1I2	1
PFKFB3	1
PTPN11	1
TLR9	1
ALDH2	0
BLM	0
CYP1B1	0
ENPP1	0

ERAP1	0
ERN1	0
KDM4A	0
MIF	0
MMP12	0
RXFP1	0
SLC1A1	0
TTR	0
TYRO3	0
XDH	0

Discussion

Reperforming network pharmacology analysis with changing selection criteria has led to different outcomes. Only HIF1A remained from the previous original analysis. It should be noted that there are no specific guidelines regarding exact thresholds or cutoffs to be followed in the selection process. It is controversial issue among previous studies which have several strategic approaches, for instance some do not consider any thresholds (El Tabaa et al., 2025, Tabaa et al., 2025, Mohsen et al., 2025, Li et al., 2026), while others consider thresholding (Li et al., 2021, Que et al., 2021), however such thresholds are highly subjective among these studies. To illustrate, Li et al. (2021) considered targets with probability >0.2, while Que et al. (2021) considered targets with probability >0.1 from swiss target prediction.

References

EL TABAA, M. M., FAHEEM, H., ELBALLAL, M. S., RASHAD, E., MOHSEN, M. & EL TABAA, M. M. 2025. The PPAR-α agonist oleoyethanolamide (OEA) ameliorates valproic acid-induced steatohepatitis in rats via suppressing Wnt3a/β-catenin and activating PGC-1α: Involvement of network pharmacology and molecular docking. *Eur J Pharmacol,* 991**,** 177306.

LI, J., ZHANG, J., KE, J., REN, Z. & FENG, C. 2026. Integrative network pharmacology and machine learning identify potential targets of indole-3-lactic acid in colorectal cancer. *PLoS One,* 21**,** e0344478.

LI, X. M., LI, M. T., JIANG, N., SI, Y. C., ZHU, M. M., WU, Q. Y., SHI, D. C., SHI, H., LUO, Q. & YU, B. 2021. Network Pharmacology-Based Approach to Investigate the Molecular Targets of Sinomenine for Treating Breast Cancer. *Cancer Manag Res,* 13**,** 1189-1204.

MOHSEN, M., ABDEL-GHANY, R. H., ELGHARBAWY, A. S., FAISAL, M. M. & ZAKARIA, E. M. 2025. Unveiling naringenin's therapeutic potential in a rat model of postmenopausal cardiovascular complications: integrated in silico and in vivo experimental approaches. *Eur J Pharmacol,* 1003**,** 177951.

QUE, W., CHEN, M., YANG, L., ZHANG, B., ZHAO, Z., LIU, M., CHENG, Y. & QIU, H. 2021. A network pharmacology-based investigation on the bioactive ingredients and molecular mechanisms of Gelsemium elegans Benth against colorectal cancer. *BMC Complement Med Ther,* 21**,** 99.

TABAA, M. M. E., HARTY, M. E. E., MOHSEN, M., RASHAD, E., NEGM, W. A., ELMORSHEDY, K. & ABU-RISHA, S. E. 2025. Integrating network pharmacology and in vivo pharmacological validation to explore the gastroprotective mechanism of Sotetsuflavone against indomethacin-induced gastric ulcer in rats: Involvement of JAK2/STAT3 pathway. *J Nutr Biochem,* 142**,** 109934.
